# Six1 is a key regulator of the developmental and evolutionary architecture of sensory neurons in craniates

**DOI:** 10.1186/1741-7007-12-40

**Published:** 2014-05-29

**Authors:** Hiroshi Yajima, Makoto Suzuki, Haruki Ochi, Keiko Ikeda, Shigeru Sato, Ken-ichi Yamamura, Hajime Ogino, Naoto Ueno, Kiyoshi Kawakami

**Affiliations:** 1 Division of Biology, Center for Molecular Medicine, Jichi Medical University, 3311-1 Yakushiji, Shimotsuke, Tochigi 329-0498, Japan; 2 Division of Morphogenesis, Department of Developmental Biology, National Institute for Basic Biology, 38 Nishigonaka, Myodaiji, Okazaki, Aichi 444-8585, Japan; 3 Faculty of Medicine, Yamagata University, 2-2-2 Iida-Nishi, Yamagata 990-9585, Japan; 4 Division of Developmental Genetics, Center for Animal Resources and Development, Institute of Resource Development and Analysis, Kumamoto University, 2-2-1 Honjo, Kumamoto 860-0811, Japan; 5 Department of Animal Bioscience, Nagahama Institute of Bio-Science and Technology, 1266 Tamura, Nagahama, Shiga 526-0829, Japan

**Keywords:** Dorsal root ganglia, Enhancer, Evolution, Neural crest cell, Rohon-Beard cell, Sensory neuron, *Six* genes

## Abstract

**Background:**

Various senses and sensory nerve architectures of animals have evolved during adaptation to exploit diverse environments. In craniates, the trunk sensory system has evolved from simple mechanosensory neurons inside the spinal cord (intramedullary), called Rohon-Beard (RB) cells, to multimodal sensory neurons of dorsal root ganglia (DRG) outside the spinal cord (extramedullary). The fish and amphibian trunk sensory systems switch from RB cells to DRG during development, while amniotes rely exclusively on the DRG system. The mechanisms underlying the ontogenic switching and its link to phylogenetic transition remain unknown.

**Results:**

In *Xenopus*, Six1 overexpression promoted precocious apoptosis of RB cells and emergence of extramedullary sensory neurons, whereas Six1 knockdown delayed the reduction in RB cell number. Genetic ablation of *Six1* and *Six4* in mice led to the appearance of intramedullary sensory neuron-like cells as a result of medial migration of neural crest cells into the spinal cord and production of immature DRG neurons and fused DRG. Restoration of SIX1 expression in the neural crest-linage partially rescued the phenotype, indicating the cell autonomous requirements of SIX1 for normal extramedullary sensory neurogenesis. Mouse *Six1* enhancer that mediates the expression in DRG neurons activated transcription in *Xenopus* RB cells earlier than endogenous *six1* expression, suggesting earlier onset of mouse SIX1 expression than *Xenopus* during sensory development.

**Conclusions:**

The results indicated the critical role of Six1 in transition of RB cells to DRG neurons during *Xenopus* development and establishment of exclusive DRG system of mice. The study provided evidence that early appearance of SIX1 expression, which correlated with mouse *Six1* enhancer, is essential for the formation of DRG-dominant system in mice, suggesting that heterochronic changes in *Six1* enhancer sequence play an important role in alteration of trunk sensory architecture and contribute to the evolution of the trunk sensory system.

## Background

**Figure 1:**
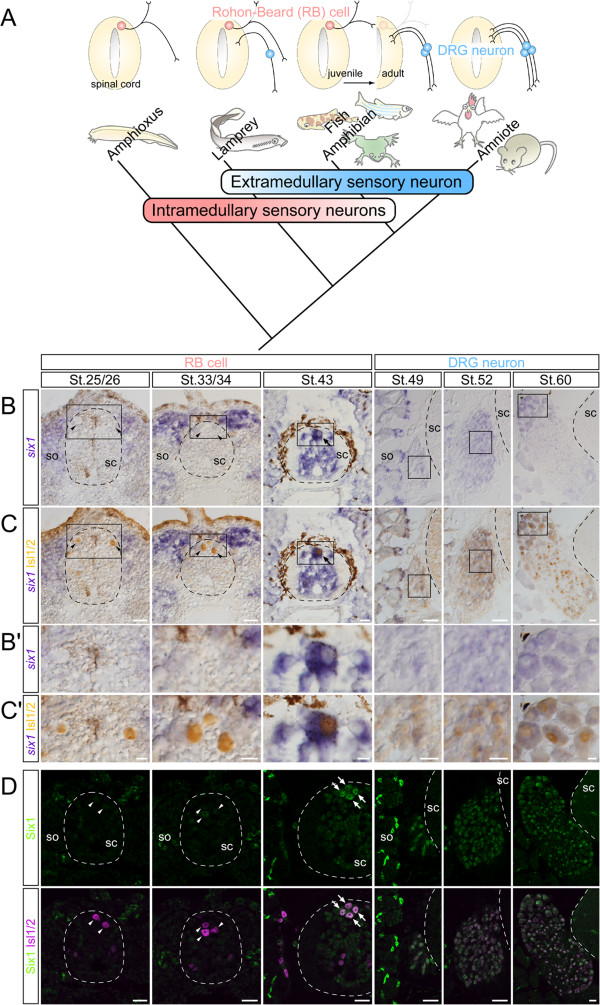
**Expression of *Xenopus *Six1 appears during transition from Rohon-Beard cells to dorsal root ganglia. (A)** Cladogram showing the succession of primary sensory neurons of chordates in the trunk. Intramedullary primary sensory neurons, called Rohon-Beard (RB) cells, dorsal cells, Retzius bipolar cells or DRiii cells (pink), are recognized in various species [5,6] - including amphioxus [13]; lampreys: *Lampetra planeri*[14], *Lampetra japonica*[15]; fish: *Lepisosteus osseus*[1], *Raja (Dipturus) batis*[16], *Scyliorhinus torazame*[17], *Danio rerio*[18]; and amphibian: *Ambystoma punctatum*[3], *Xenopus laevis*[4], *Rana pipiens*[19], *Rana catesbeiana*[20], *Ceratophrys ornata*[21], *Eleutherodactylus coqui*[22] - but not in amniotic vertebrates [6,7]. Craniates have extramedullary sensory neurons (blue) that are derived from neural crest cells and form dorsal root ganglia (DRG). **(B-D)** Distribution of Six1 mRNA and protein in the trunk region of *Xenopus* embryos (dorsal side: top of transverse sections). Developmental stages are indicated at top line. Arrowheads: Six1-negative RB cells; arrows: Six1-positive RB cells. **(B, C)***In situ* hybridization of *six1* (purple) is followed by immunostaining of Isl1/2 (orange), marking RB cells and DRG neurons. **(B’, C’)** Magnified views of the areas indicated by the rectangles in **B** and **C**. **(D)** Detection of Six1 (green) and Isl1/2 (magenta) by immunofluorescence in sections adjacent or alternate to those in **B** and **C**. Dashed lines demarcate the position of the spinal cord. sc, spinal cord; so, somite. Scale bars: 25 μm **(B-D)** and 10 μm **(B’, C’)**.

Trunk sensory neurons convey somatic and visceral information to the central nervous system (CNS). Rohon-Beard (RB) cells are known to mediate the sensory pathway in fish and amphibian larvae [1-5]; however, this cell type has not been identified in avian and mammalian species [6,7] (Figure 1A). In fish and amphibians, RB cells are located in the dorsal part of the spinal cord and have peripheral and central neurites. The peripheral neurites innervate the skin of the trunk, while the central neurites descend and ascend over several segments within the spinal cord, ultimately reaching the brainstem [2,8]. At later larval stages, RB cells undergo cell death by apoptosis. Concomitantly, neural crest cell (NCC)-derived extramedullary sensory ganglia (dorsal root ganglia; DRG) develop and begin to process mechanosensory inputs [9]. At present, evidence suggests that the body organization of cephalochordate Amphioxus reflects the early primitive condition of chordate. Although it lacks extramedullary sensory neurons, equivalent to DRG neurons, it has two types of intramedullary neurons comparable to RB cells of anamniotes, Retzius bipolar cells in larvae [10,11] and DRiii cells in adult [12]. In agnathan lampreys, both RB cells and extramedullary sensory neurons are present, but their spinal roots are primitive and asymmetrical [7]. Due to the ontogenic transition in anamniotes from RB cells to DRG neurons and phylogenetic disappearance of RB cells in amniotes, intramedullary cells are regarded as the prototype of sensory neurons in chordates (Figure 1A).

Both RB cells and NCCs arise from the cells present at the border of the neural- and non-neural ectoderm and both require similar inductive signals including bone morphogenetic proteins [23]. Notch/Delta signaling alternatively determines their fates; it promotes RB cell fate at the expense of NCC-derived cell types [24,25]. Whereas NCCs undergo epithelial-to-mesenchymal transition and migrate to their final destinations, including DRG [26], RB cells migrate medially from the border and are finally incorporated into the medulla [27-29]. RB cells extend their neurites and mediate the sensory circuits required for rhythmic swimming and predator avoidance [30,31]. At the approach of metamorphosis, the functions of RB cells are superseded by DRG neurons, and the death of RB cells is induced by the attenuation of TrkC1/NT-3 signaling [32]. Although previous studies shed light on the induction, specification and differentiation processes of RB cells in zebrafish and *Xenopus*[33], the molecular mechanisms underlying the replacement of RB cells with DRG neurons remain largely unknown. Unveiling such mechanisms should enhance our understanding of the evolution of sensory architecture, particularly the emergence of DRG in craniates and subsequent disappearance of RB cells in amniotes.

In the cranial region, the *Six1* homeobox gene is considered the main player in the genesis of sensory organs: loss of function of *Six1* causes severe defects in various sensory organs that originate from the cranial sensory placodes in zebrafish [34,35], *Xenopus*[36,37], mouse [38-43] and humans [44-46]. The expression of *Six4*, another *Six* family gene, shows a similar pattern, and simultaneous loss of both genes aggravated the defects in mice [47-49]. While *Six1* and *Six4* are also expressed in DRG in the trunk [40,50], the functional significance of the two genes in the development of DRG neurons and RB cells has not been explored.

In the present study, we found that Six1 is expressed in *Xenopus* RB cells just before apoptotic cell death. Experiments involving Six1 overexpression and knockdown demonstrated that Six1 is a key molecule for the ontogenic transition of RB cells to DRG neurons during development. Moreover, in mice, SIX1 and SIX4 were essential for the normal development of DRG, and loss of both genes allowed the emergence of intramedullary sensory neuron-like cells, as a result of immigration of NCCs into the spinal cord. We also investigated the molecular basis of the differential onset of *Six1* expression in both *Xenopus* and mouse. The results showed changes in the activity of a conserved enhancer. Based on these results, we suggest that heterochronic shift in *Six1* expression contributes to phylogenetic transition in the architecture of sensory neurons.

## Results

### Six1 expression is turned on immediately before the onset of *Xenopus* Rohon-Beard cell apoptosis

To investigate the functions of both Six1 and Six4 in the development of trunk sensory neurons, we first examined their expression patterns in *Xenopus* embryos (Figure 1B,C). RB cells are characterized by their large size and dorsal location in the spinal cord and their nuclei are positive for Isl1/2(Islet1/2) [51,52]. No mRNA or protein expression of Six1 and Six4 was observed in RB cells at stages (St.) 16/17, 25/26 and 33/34 (arrowheads in Figure 1B,C; Additional file 1). However, Six1 mRNA and protein expression was recognized in RB cells at St. 43 (arrows in Figure 1B,C). The majority of DRG neurons (identified by their Isl1/2-positive nuclei and location within the dorsal root) were positive for Six1 from St. 49 to 60. The number of RB cells started to decrease at around St. 46 due to apoptosis [9]. These findings indicate that Six1 expression appears before apoptosis of RB cells, and persists during DRG formation in *Xenopus* development, although both are trunk sensory neurons.

### Six1 controls the transition from Rohon-Beard cells to dorsal root ganglia during *Xenopus* development

**Figure 2:**
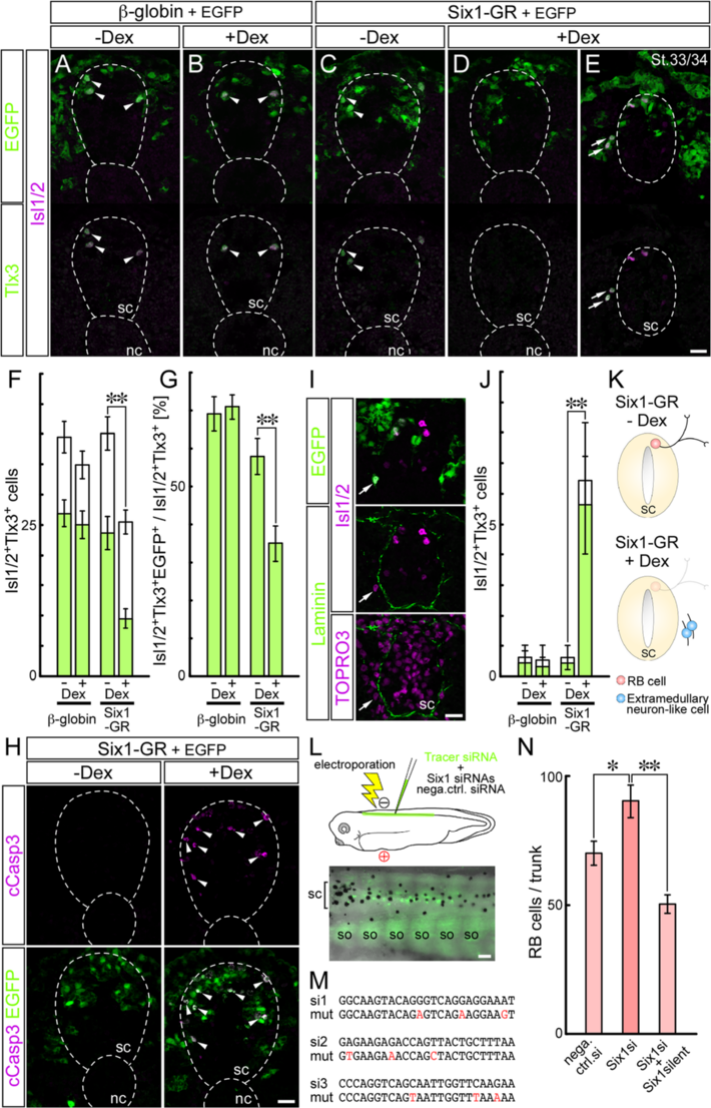
**Six1 mediates the developmental transition of trunk sensory neurons during *Xenopus *development. (A-K)** Earlier expression of Six1 reduces the number of Rohon-Beard (RB) cells and promotes differentiation of extramedullary sensory neurons in *Xenopus.***(A-E)** The combinations of injected mRNAs and treatment with dexamethasone (Dex) are indicated at the top. Arrowheads: enhanced GFP (EGFP)-positive RB cells, which are marked with both Isl1/2 (magenta) and Tlx3 (green) in the spinal cord (sc) at St. 25/26 **(A-D)**. Arrows: EGFP-, Isl1/2- and Tlx3-triple-positive cells outside sc at St. 33/34 **(E)**. **(F)** Quantification of RB cells and **(G)** percentage of EGFP-positive RB cells in 250 μm of the thoracic level at St. 25/26. White bars: total number of RB cells, green bars: EGFP-positive cells. **(H)** EGFP- and cleaved CASP3 (cCasp3)-double-positive cells (arrowheads) are noted in Six1-GR + Dex. **(I)** In Six1-GR + Dex embryos, Isl1/2- and EGFP-double positive cells (arrow) are located outside sc. Laminin (green): sc outline, TOPRO3 (magenta): nuclei. **(J)** Quantification of Isl1/2- and Tlx3-double positive cells outside sc. White bars: total number of cells, green bars: EGFP-positive cells. **(K)** Schematic representation of the results. Activation of Six1 reduced the number of RB cells (pink) in sc and enhanced the differentiation of DRG neuron-like cells (blue) outside sc. **(L-N)** siRNA mediated knockdown of Six1 increased the number of RB cells. **(L)** Top: schematic representation of electroporation of *Xenopus* embryo. Bottom: merged picture of epifluorescence and bright field in obliquely dorsal view of the trunk at St. 45. Fluorescein isothiocyanate-labeled control small interfering RNA (siRNA) persists in the dorsal sc. **(M)** si1, si2 and si3: siRNA targeted sequences in *six1*; mut: three silent mutations, each corresponding to siRNA targeted regions in mutated *six1*. **(N)** Number of Isl1/2-positive RB cells in the trunk (level in somite (so) pairs 1 to 9) at St. 45/46. Data are mean ± standard error of the mean. *p <0.01; **p<0.001. Dashed lines demarcate the position of sc and notochord (nc). Scale bars: 100 μm **(L)** and 25 μm **(A-K,M,N)**.

The above findings suggested that Six1 is a key regulator of apoptotic death of RB cells and the development of DRG during the transition of sensory neurons in *Xenopus*. To test this notion, we investigated the effects of Six1 overexpression and knockdown (Figure 2). To avoid any disturbance of early embryonic development, especially placodal development and early neuronal differentiation [36], we utilized a steroid hormone-inducible system [53]. We generated mRNA encoding *Xenopus* Six1 fused to the glucocorticoid receptor ligand-binding domain (GR) (Six1-GR) and injected it into V1.2 blastomeres at the 16-cell stage. During normal development, the blastomeres give rise to the dorsal spinal cord and ectoderm, including RB cells [54] (Additional file 2A). Six1-GR was then activated by adding dexamethasone (Dex) at St. 16/17, at the time when the majority of RB cells had already exited the cell cycle [55]. The embryos were analyzed at St. 25/26.

In all experiments, enhanced GFP (EGFP) mRNA was co-injected to trace the distribution and lineage of the injected cells. RB cells, which are positive for both Isl1/2 and Tlx3 (XHox11L2) [56], were effectively labeled with EGFP in embryos injected with mRNA encoding β-globin as a control (Figure 2A). Dex-treatment did not alter the distribution or number of RB cells, or the proportion of EGFP-positive RB cells in embryos injected with β-globin mRNA (39.5 ± 2.6 cells in -Dex, n = 12; 34.9 ± 2.3 cells in + Dex, n = 11; Figure 2B,F). The number of RB cells in embryos injected with Six1-GR mRNA decreased significantly to 25.5 ± 2.0 cells (n = 17, *p* = 0.0002) after Dex-treatment, compared with Dex-untreated control (40.1 ± 2.8 cells, n = 16; Figure 2C,D,F) and with β-globin mRNA-injected embryos (Figure 2F). The fraction of EGFP-positive RB cells was also reduced by Dex-treatment (Figure 2G). To determine whether the observed reduction in RB cells was due to apoptosis, which is known to occur later during normal development, we performed immunostaining for cleaved CASP3 (Caspase-3). No signal was detected in the EGFP-positive cells within the dorsal spinal cords of embryos injected with Six1-GR mRNA and untreated, whereas the signal was clearly detected in Dex-treated embryos at St. 25/26 (Figure 2H). These findings suggest that precocious expression of Six1 accelerates the disappearance of RB cells, at least in part, through the induction of apoptosis.

Surprisingly, at St. 33/34, Isl1/2- and Tlx3-double-positive cells were observed outside the spinal cord in the Six1-GR-overexpressing and Dex-treated embryos (7.2 ± 1.2 cells, n = 13), but rarely in the control embryos (1.1 ± 0.6 cells in β-globin -Dex, n = 9; 0.4 ± 0.3 cells in β-globin + Dex, n = 9; 1.0 ± 0.3 cells in Six1-GR -Dex, n = 12; Figure 2E,I,J). Importantly, most of these cells were positive for EGFP (93.5 ± 3.3%), suggesting cell-autonomous effect of Six1-GR activation. Although Isl1/2 and Tlx3 are markers for both DRG neurons and RB cells (Additional file 2B), the extramedullary and ventrolateral location of Isl1/2- and Tlx3-double-positive cells suggests that these sensory neuron-like cells are closely related to DRG neurons (Figure 2K). Taken together, these findings suggest that overexpression of Six1 promotes premature differentiation of extramedullary neurons in *Xenopus* embryos.

To evaluate the effects of loss-of-function of Six1 on the transition of trunk sensory neurons, small interfering RNAs (siRNAs) were introduced into the dorsal spinal cord through electroporation (Figure 2L). We generated three different siRNAs for Six1 (Figure 2M). The knockdown efficacy of the mixture of Six1 siRNAs was verified in HEK293 cell line (Additional file 2C). To avoid disturbance of early development by Six1 siRNAs, electroporation was performed on St. 34/35 embryos, before the onset of cell death [9]. Because the maximum number of RB cells appears at St. 41 and the number falls thereafter [9] (Additional file 2D), counting of RB cells in the trunk region (in somite pairs 1 to 9) was conducted at St. 45/46 (Figure 2N). The number of RB cells significantly increased from 70.1 ± 4.6 cells in the control embryos (n = 21) to 90.5 ± 6.4 cells in embryos electroporated with the Six1 siRNAs (n = 15, *p* = 0.01; Figure 2N). To validate the specificity of the Six1 siRNAs, we performed a rescue experiment by employing Six1-containing silent mutations in the siRNA target sequences (Figure 2M), which was resistant to our siRNAs (Additional file 2C). Co-electroporation of Six1 siRNAs with the mutated *six1* mRNA reversed the knockdown effect of siRNAs (50.2 ± 3.6 cells, n = 22, *p* = 0.000001) (Figure 2N), confirming that the knockdown of Six1 itself causes a delay in the reduction of the number of RB cells.

Taken together, the above findings indicate that early expression of Six1 leads to a decrease in the number of RB cells, while it promotes extramedullary sensory neurogenesis. Additionally, knockdown of Six1 leads to delay in the reduction of RB cell number. Based on these results, it is conceivable that Six1 is involved in purging RB cells and promoting the formation of DRG during *Xenopus* development.

### SIX1 and SIX4 control the development of mouse dorsal root ganglia

**Figure 3:**
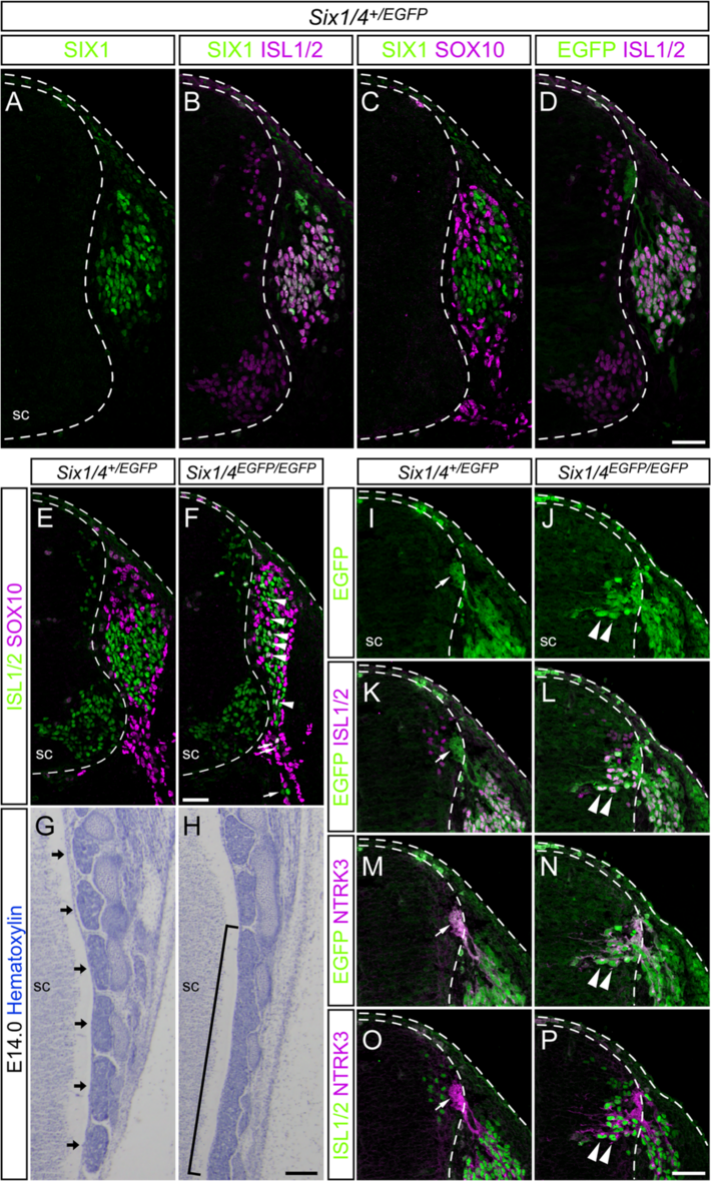
**SIX1 is crucial during mouse dorsal root ganglia development. (A-D)** Immunofluorescence of SIX1 (green), neuronal marker ISL1/2 (magenta) and glial marker SOX10 (magenta) in dorsal root ganglia (DRG) of embryonic day (E) 11.5 *Six1/4*^*+/EGFP*^ embryos, in which the *Six1*-locus directs the expression of enhanced GFP (EGFP; green). **(E-P)** Abnormalities in *Six1/4*^*EGFP/EGFP*^ DRG. **(E,F)** Arrowheads: ISL1/2 (green)- and SOX10 (magenta)-double-positive cells; arrows: ventrally located ISL1/2-positive cells **(F)**, which were never observed in *Six1/4*^*+/EGFP*^ at E11.5 **(E)**. **(G,H)** Coronal sections of lumbar region at E14.0 (top: rostral side). DRG are fused over several segments in *Six1/4*^*EGFP/EGFP*^ embryos (square bracket in **H**), but segmented in *Six1/4*^*+/EGFP*^ (arrows in **G**). **(I-P)** Transverse sections of dorsal spinal cords (sc) at E11.5. Arrowheads: intramedullary EGFP (green)-, ISL1/2 (magenta)- and NTRK3 (magenta)-triple-positive cells only in *Six1/4*^*EGFP/EGFP*^; arrows: EGFP-positive afferents at the dorsal root entry zone. Dashed lines demarcate the position of ectoderm and sc. Scale bars: 200 μm **(G,H)** and 50 μm **(A-F,I-P)**.

We next examined the roles of SIX1 and SIX4 in the development of sensory neurons in the trunk of mice. Immunofluorescence staining with specific SIX1 and SIX4 antibodies [39,48] (Additional file 3A) showed similar distribution patterns for SIX1 and SIX4 proteins in the DRG at embryonic day (E) 11.5 (Additional file 3B). SIX1 was located in approximately 50% of the ISL1/2-positive neurons (Figure 3A,B), but not in SOX10-positive undifferentiated NCCs or glia (Figure 3C). We described previously the generation of *Six1* and *Six4* knockout alleles that harbor *EGFP* and *LacZ* genes, respectively [48,57]. Because heterozygous embryos harboring the two knockout alleles (*Six1*^*+/-*^/*Six4*^*+/-*^, denoted as *Six1/4*^*+/EGFP*^) did not exhibit obvious anomalies in DRG development (Figure 3A-C), we used them to examine *Six1* expression by monitoring green fluorescence. EGFP-positive soma in *Six1/4*^*+/EGFP*^ embryos was mostly positive for ISL1/2 immunofluorescence (Figure 3D), indicating activation of the *Six1* locus in the majority of ISL1/2-positive neurons at some point during differentiation, because EGFP protein can reside in cells for a longer period than SIX1 protein. These observations point to the potential role of SIX1 in differentiation of NCC into DRG neurons.

The DRG of double-knockout embryos (*Six1*^*-/-*^*/Six4*^*-/-*^, denoted as *Six1/4*^*EGFP/EGFP*^) were smaller and flat mediolaterally compared to those of the wild type and *Six1/4*^*+/EGFP*^ at E11.5 (Figure 3E,F). In addition, ISL1/2-positive neurons were dispersed and emigrated ventrally compared with those in *Six1/4*^*+/EGFP*^ (Figure 3E,F). Furthermore, ISL1/2- and SOX10-double-positive cells, which are reminiscent of immature NCCs, were frequently observed (n = 19, Figure 3F), whereas such cells were rarely recognized in wild type and *Six1/4*^*+/EGFP*^ (Figure 3E). Finally, the lumbar DRG was found to fuse later in the development of *Six1/4*^*EGFP/EGFP*^ (Figure 3G,H). Considered together with the lack of significant developmental anomalies in DRG in *Six1* and *Six4* single-knockout embryos [50,58], these findings suggest mutually compensatory roles for SIX1 and SIX4 in the differentiation and migration of DRG cells and gangliogenesis.

The axonal bundle at the dorsal root entry zone (DREZ) [59] was also positive for EGFP in *Six1/4*^*+/EGFP*^ at E11.5 (arrows in Figure 3I,K). This was owing to intracellular diffusion of the long-lasting EGFP protein, indicating that the sensory afferents originated from SIX1-positive neurons in DRG. Surprisingly, EGFP- and ISL1/2-double-positive cells were noted inside the spinal cord near the DREZ of *Six1/4*^*EGFP/EGFP*^ (n = 19, Figure 3J,L), whereas these cells were hardly observed in *Six1/4*^*+/EGFP*^ (n = 17, Figure 3I,K). The EGFP- and ISL1/2-double-positive cells in *Six1/4*^*EGFP/EGFP*^ also showed immunofluorescence signal for NTRK3 (TrkC) (n = 6, Figure 3N,P), a marker of proprioceptive neurons of the DRG [60]. By contrast, the NTRK3 signal was observed only in the axonal bundle of DREZ in the spinal cords of *Six1/4*^*+/EGFP*^ (n = 5, Figure 3M,O). These findings suggest the novel roles for SIX1 and SIX4 in the development of DRG and preclusion of intramedullary sensory neurons.

### SIX1 prevents the appearance of sensory neurons in mouse spinal cord

**Figure 4:**
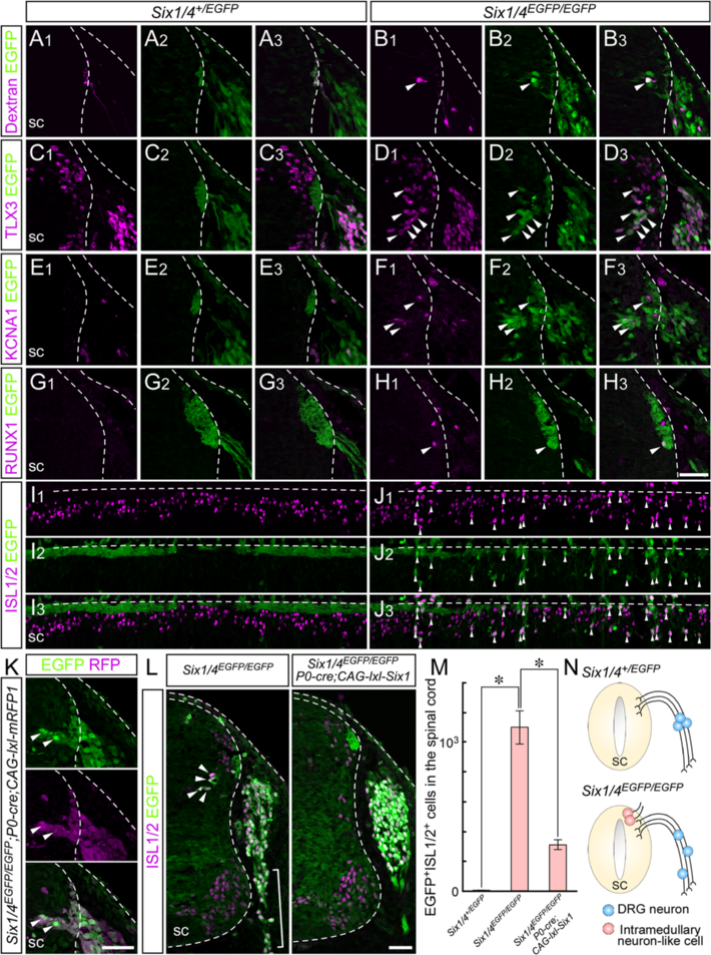
**Appearance of intramedullary sensory neuron-like cells in *Six1/4***^***EGFP/EGFP ***^**mice. (A-H)** Magenta represents rhodamine-dextran injected outside the spinal cord (sc) **(A,B)**, TLX3 **(C,D)**, KCNA1 **(E,F)**, RUNX1 **(G,H)** and **(I,J)** ISL1/2, which are detected in intramedullary enhanced GFP (EGFP)-positive cells (green) in *Six1/4*^*EGFP/EGFP*^ spinal cord. Arrowheads: double-positive cells. **(K)** Intramedullary EGFP-positive cells (green) are labeled with neural crest cell (NCC) lineage-specific fluorescence of monomeric RFP (magenta). **(L)** EGFP (green)- and ISL1/2 (magenta)-double-positive intramedullary cells disappear with NCC-specific restoration of SIX1. **(M)** Number of EGFP- and ISL1/2-double-positive cells in the spinal cord at the level of somite 21 to 26. **(N)** Schematic representation of dorsal root ganglion (DRG) neurons (blue) and intramedullary neuron-like cells (pink) in *Six1/4*^*+/EGFP*^ and *Six1/4*^*EGFP/EGFP*^. **A-F** and **I-L** are on embryonic day **(E)**11.5; **G** and **H** are on **E** 12 . **A-H, K** and **L** are transverse sections (top: dorsal side); **I** and **J** are coronal sections (top: lateral side). Data are mean ± standard error of the mean. *p <0.001. Dashed lines demarcate the position of ectoderm and sc. Scale bars: 50 μm.

To define the properties of intramedullary EGFP-positive cells in *Six1/4*^*EGFP/EGFP*^, we first assessed the projection of neurites. The retrograde tracer rhodamine-dextran was injected into a region outside the spinal cord in E11.5 embryos and subsequently observed in EGFP-positive soma in the spinal cords of *Six1/4*^*EGFP/EGFP*^ (n = 3, Figure 4B). By contrast, the tracer was noted only in the sensory axon bundle at the DREZ of *Six1/4*^*+/EGFP*^ (n = 3, Figure 4A). These results indicate that these EGFP-positive cells in *Six1/4*^*EGFP/EGFP*^ extend their processes outside the spinal cord. In the thoracolumbar region of wild-type mouse spinal cord, only somatic motor neurons and preganglionic motor column cells with projections to the sympathetic ganglia send axons from the inside of the spinal cord to the outside, through the ventral root [61]. The atypical projection of the intramedullary EGFP-positive cells of *Six1/4*^*EGFP/EGFP*^ embryos suggests resemblance to the intramedullary sensory neurons, RB cells, of anamniotes.

*Xenopus* RB cells are Tlx3-positive (see Figure 2). The majority of intramedullary EGFP-positive cells in *Six1/4*^*EGFP/EGFP*^ were positive for TLX3 (n = 6, Figure 4D). The DRG neurons and subsets of differentiated interneurons in the dorsal spinal cord were also TLX3-positive, as reported previously [62] (Figure 4C), however, these cells appeared normal in *Six1/4*^*EGFP/EGFP*^ (n = 6, Figure 4C,D). The transcript of a Shaker-like potassium channel Kcna1 (Kv1.1) was detected previously in *Xenopus* RB cells [63], and we confirmed the protein localization of Kcna1 in RB cells by immunohistochemical analysis (Additional file 2B). In E11.5 *Six1/4*^*EGFP/EGFP*^ embryos, a subset of intramedullary EGFP-positive cells was positive for KCNA1 (n = 3, Figure 4F), although no such cells were observed in the dorsal spinal cord of *Six1/4*^*+/EGFP*^ embryos (Figure 4E). Runt-related transcription factors, Runx1 and Runx3, are expressed in *Xenopus* RB cells and Runx1 is critical for the development of RB cells [64,65]. The majority of intramedullary EGFP-positive cells in *Six1/4*^*EGFP/EGFP*^ were positive for both proteins (n = 3, Figure 4H; Additional file 4A), although no such cells were recognized in *Six1/4*^*+/EGFP*^ spinal cord (n = 5, Figure 4G). RB cells are distributed in a non-segmental manner [8,66], whereas DRG show segmental and symmetrical organization along the body axis. The intramedullary EGFP-positive cells of *Six1/4*^*EGFP/EGFP*^ never showed any distinguishable segmental arrangements (Figure 4I,J). Considered together, these findings suggest that intramedullary EGFP-positive cells have substantial common features with the amphibian RB cells.

The sensory neurons in DRG originate from the NCC. Next, we examined whether the EGFP-positive cells in the spinal cord of *Six1/4*^*EGFP/EGFP*^ originate from NCC by employing a genetic strategy to label NCC lineage. By crossing *P0-Cre* mice, in which Cre recombinase is expressed in tissues derived from NCCs (*P0-Cre*) [67], with a transgenic mouse line harboring *CAG-loxP-STOP-loxP-mRFP1* cassette (*CAG-lxl-mRFP1*), the neural crest lineage can be visualized by the red fluorescence of monomeric RFP (mRFP). Virtually all EGFP-positive intramedullary cells in the *Six1/4*^*EGFP/EGFP*^*;P0-Cre;CAG-lxl-mRFP1* embryos were labeled with mRFP1, indicating that they originated from the neural crest (n = 3, Figure 4K).

In the mouse trunk, SIX1 and SIX4 are expressed in somites and the mesenchyme [40,50] in addition to the DRG. To determine whether the emergence of intramedullary EGFP-positive cells in *Six1/4*^*EGFP/EGFP*^ embryos was caused by loss of functions of SIX1 and SIX4 in NCCs, we restored the expression of SIX1 only in NCC derivatives. For this purpose, we generated a transgenic mouse line harboring *CAG-loxP-STOP-loxP-Six1* cassette (*CAG-lxl-Six1*) and crossed it with *P0-Cre* mice. The number of EGFP- and ISL1/2-double-positive cells was markedly reduced in the spinal cords of *Six1/4*^*EGFP/EGFP*^*;P0-Cre;CAG-lxl-Six1* embryos (n = 4, Figure 4L). The abnormal morphology and organization of *Six1/4*^*EGFP/EGFP*^ DRG were also rescued and the DRG resembled those of *Six1/4*^*+/EGFP*^ (Figure 4L, compare with Figure 3). The number of EGFP- and ISL1/2-double-positive cells in the spinal cord at the level of somites 21 to 26 was significantly higher in *Six1/4*^*EGFP/EGFP*^ embryos (1,097.5 ± 113.6 cells/embryo, n = 4, *p* = 0.00007), compared to *Six1/4*^*+/EGFP*^ embryos (4.0 ± 1.3 cells/embryo, n = 4; Figure 4M). NCC lineage-specific restoration of SIX1 expression markedly reduced the number of these cells to 309.8 ± 33.1 cells/embryo in *Six1/4*^*EGFP/EGFP*^*;P0-Cre;CAG-lxl-Six1* mice (n = 4, *p* = 0.0006; Figure 4M). These results suggest that the cell-autonomous function of SIX1 in the NCC primarily suppresses the developmental program responsible for the generation of intramedullary sensory neuron-like cells during mouse development.Taken together, these findings highlight the importance of SIX1 and SIX4 in NCC-lineage in the formation of DRG and preclusion of intramedullary sensory neuron-like cells in mice (Figure 4N).

### Medial migration of neural crest cells results in intramedullary sensory neuron-like cells

**Figure 5:**
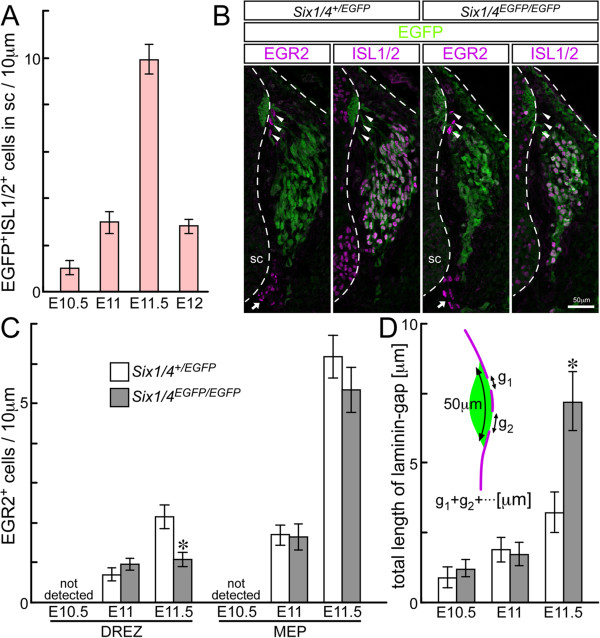
**Appearance of intramedullary sensory neuron-like cells in *Six1/4***^***EGFP/EGFP ***^**mice precedes disorganization of the dorsal root entry zone. (A)** Number of enhanced GFP (EGFP)- and ISL1/2-double-positive cells in 10 μm sections of embryonic day (E) 10.5 to 12 *Six1/4*^*EGFP/EGFP*^ thoracolumbar spinal cord. **(B)** EGR2-positive cells (magenta) at the dorsal root entry zone (DREZ) (arrowheads) and at the motor exit point (MEP) (arrows) do not overlap with EGFP (green) and show comparable distribution in E11.5 embryos of *Six1/4*^*+/EGFP*^ and *Six1/4*^*EGFP/EGFP*^. ISL1/2 (magenta) marks dorsal root ganglion neurons. **(C)** Number of EGR2-positive cells in 10 μm sections of E10.5 to E11.5 at the DREZ and MEP of *Six1/4*^*+/EGFP*^ and *Six1/4*^*EGFP/EGFP*^. **(D)** Total length of laminin gaps in 50-μm length of basal lamina covering the primordium of the dorsal funiculus. In all measurements, at least five sections from three embryos were used per genotype. Data are mean ± standard error of the mean. *p <0.005. Dashed lines demarcate the position of ectoderm and spinal cord (sc). Scale bar: 50 μm.

**Figure 6:**
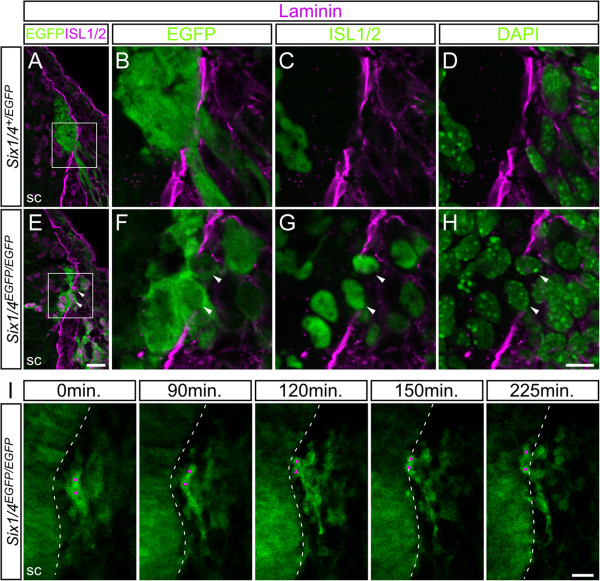
**Medial migration of intramedullary sensory neuron-like cells in *Six1/4***^***EGFP/EGFP ***^**mice. (A-H)** Abnormalities in the *Six1/4*^*EGFP/EGFP*^ dorsal root entry zone (DREZ). **(B-D)** and **(F-H)** show close-up of the DREZ (indicated with rectangles) in *Six1/4*^*+/EGFP*^**(A)** and *Six1/4*^*EGFP/EGFP*^**(E)**. ISL1/2 (magenta/green)- and enhanced GFP (EGFP; green)-double positive cells (arrowheads) are located across the basal lamina, which is demarcated by the immunofluorescence signal of laminin (magenta). The position of nuclei is visualized with DAPI (green). **(I)** Time-lapse imaging of EGFP-positive cells on the slice culture of embryonic day 11 *Six1/4*^*EGFP/EGFP*^ lumbar region. Top line shows elapsed time of observation. See also Additional file 5. Dashed lines demarcate the position of the spinal cord (sc). Scale bars: 25 μm **(A,E,I)** and 10 μm **(B-D,F-H)**.

To determine the mechanism and source of intramedullary sensory neuron-like cells, we examined their ontogeny. EGFP-positive intramedullary cells were first observed at E10.5 at the level of the thoracolumbar region in *Six1/4*^*EGFP/EGFP*^ embryos, and their number reached peak level at E11.5 (Figure 5A). The basal lamina marked with laminin-staining was often interrupted by the presence of EGFP- and ISL1/2-double-positive cells at the DREZ in E11 to 11.5 *Six1/4*^*EGFP/EGFP*^ embryos (n = 8, Figure 6E-H), although the gaps of basal lamina were normally occupied by axons originating from DRG neurons in *Six1/4*^*+/EGFP*^ (n = 17, Figure 6A-D). Time-lapse live imaging analysis of slice cultures of the E11 *Six1/4*^*EGFP/EGFP*^ embryos at lumbar level indicated that EGFP-positive cells migrated medially toward the spinal cord and finally into it (n = 5, Figure 6I; Additional file 5). Taken together with the results of labeling and rescue analysis using *P0-Cre* (Figure 4K-M), medial migration of NCCs into the spinal cord seems a plausible mechanism for the emergence of intramedullary EGFP-positive cells in *Six1/4*^*EGFP/EGFP*^.

### Lack of dorsal root entry zone disruption before emergence of intramedullary sensory neuron-like cells

To explore the mechanism underlying the emergence of sensory neuron-like cells in the mouse spinal cord, we examined the boundary structure between the CNS and peripheral nervous system (PNS) on the surface of the spinal cord.

Boundary cap (BC) cells are known to maintain the integrity of the boundary structure between the CNS and PNS. Specifically, BC cells prevent the emigration of motor neurons at the ventral part of the spinal cord (the MEP) and of glial cells to the periphery at the DREZ [68,69]. BC cells can be identified by the expression of EGR2 (KROX20) [70,71]. EGR2-positive nuclei did not overlap with EGFP fluorescence in either *Six1*/*4*^*+/EGFP*^ or *Six1*/*4*^*EGFP/EGFP*^ embryos at E11.5 (Figure 5B), and the distribution of EGR2-positive cells located at the DREZ and MEP was not apparently different between *Six1/4*^*+/EGFP*^ and *Six1/4*^*EGFP/EGFP*^ (arrowheads and arrows in Figure 5B, respectively). At E10.5, no EGR2-positive cells were observed at the DREZ (assayed in 14 embryos with 30 to 35 pairs of somites, Figure 5C), although they were observed in the cervical region [72]. EGR2-positive cells were first detected at the level of the thoracolumbar region at E11 (n = 5, Figure 5C) after the appearance of intramedullary EGFP-positive cells at E10.5. At E11, the numbers of EGR2-positive cells at both the DREZ and MEP were not different between *Six1/4*^*+/EGFP*^ and *Six1/4*^*EGFP/EGFP*^. At E11.5, the number of EGR2-positive cells at the DREZ of *Six1/4*^*EGFP/EGFP*^ (1.1 ± 0.2 cells/10 μm, n = 5, *p* = 0.004) was approximately half that of *Six1/4*^*+/EGFP*^ (2.2 0.3 cells/10 μm, n = 6), although the number was similar in MEP. These findings indicate that the emergence of intramedullary EGFP-positive cells at the level of the thoracolumbar region precedes that of BC cells in *Six1/4*^*EGFP/EGFP*^ embryos. At E11.5, when intramedullary EGFP-positive cells were largest in number in *Six1/4*^*EGFP/EGFP*^ embryos, the number of BC cells at the DREZ of *Six1/4*^*EGFP/EGFP*^ was smaller compared with that of *Six1/4*^*+/EGFP*^.

We also investigated the distribution of laminin, a major component of the basal lamina, to evaluate the integrity of the boundary structure between the CNS and PNS at the DREZ. Gaps in the laminin layer usually mark the site of penetration of dorsal roots from DRG neurons into the spinal cord (Figure 6A-D) [73]. In *Six1/4*^*EGFP/EGFP*^ embryos, intramedullary EGFP-positive cells could originate from the cells that pass through the gaps and invade the spinal cord in the presence of possible disruption of the border. Gaps in the immunofluorescence signal of laminin were observed in the DREZ of both *Six1/4*^*+/EGFP*^ and *Six1/4*^*EGFP/EGFP*^ embryos (Figure 5D). At E11.5, the total length of laminin gaps in 50-μm length of basal lamina covering the DREZ was significantly greater in *Six1/4*^*EGFP/EGFP*^ (7.2 ± 1.1 μm, n = 4, *p* = 0.004) than *Six1/4*^*+/EGFP*^ (3.2 ± 0.7 μm, n = 4). The difference was caused by the larger number of laminin gaps rather than longer individual gaps in the *Six1/4*^*EGFP/EGFP*^ DREZ compared with *Six1/4*^*+/EGFP*^ (Additional file 6). However, the total length of laminin gaps was comparable in the DREZ of *Six1/4*^*+/EGFP*^ and *Six1/4*^*EGFP/EGFP*^ at earlier developmental stages of E10.5 and E11, at which intramedullary EGFP-positive cells were already present. These findings suggest that the number of laminin gaps at the DREZ increased after the emergence of intramedullary EGFP-positive cells in *Six1/4*^*EGFP/EGFP*^ embryos.

Considered together, the above findings suggest no apparent change in the boundary structure at the DREZ before the emergence of intramedullary sensory neuron-like cells in *Six1/4*^*EGFP/EGFP*^ embryos, and that the number of BC cells decreased while that of laminin gaps increased after the emergence of intramedullary cells. Thus, the emergence of sensory neuron-like cells in the spinal cord of *Six1/4*^*EGFP/EGFP*^ embryos could be due to translocation of NCCs into the spinal cord, and the most probable mechanism for such translocation is a change in the intrinsic properties of NCCs.

### Mouse *Six1* enhancer is activated earlier than *Xenopus* enhancer in Rohon-Beard cells

**Figure 7:**
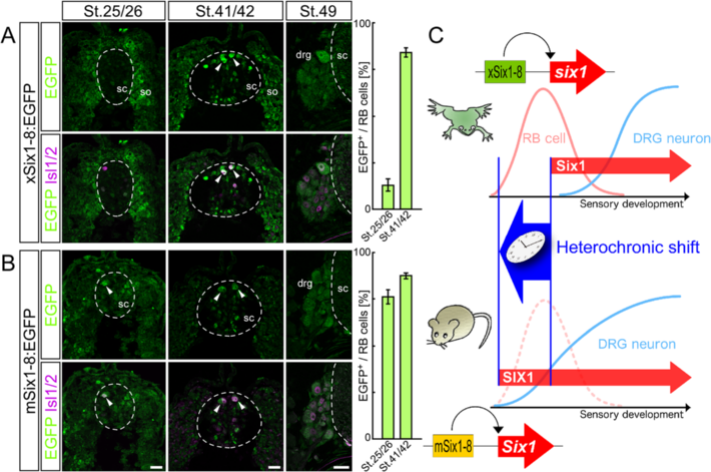
**Mouse *Six1 *enhancer for dorsal root ganglion neurons directs expression in Rohon-Beard cells earlier than *Xenopus *enhancer. (A)***Xenopus* enhancer directed the expression of enhanced GFP (EGFP; green) in Rohon-Beard (RB) cells (magenta, labeled by Isl1/2) not at Stages (St.) 25/26, but at St. 41/42, and in dorsal root ganglion (DRG) neurons at St. 49. **(B)** Mouse enhancer directed expression of EGFP in RB cells at St. 25/26 and 41/42, and in DRG neurons at St. 49. For **A** and **B**, bar graphs show the percentage of EGFP-positive RB cells in 250 μm of the thoracic level. Data are mean ± standard error of the mean. Arrowheads: EGFP- and Isl1/2-double-positive RB cells; dashed lines demarcate the position of the spinal cord (sc); so, somite; drg, dorsal root ganglia. Scale bars: 25 μm. **(C)** Schematic representation of timing of Six1 expression in *Xenopus* and mouse. *Xenopus six1* enhancer for sensory neurons (xSix1-8) directs the expression of Six1 (red arrow) to alter the sensory system from RB cells (pink line) to DRG neurons (blue line). In mouse, *Six1* enhancer (mSix1-8) mediates SIX1 expression earlier during trunk sensory development. This altered timing of SIX1 expression inhibits the development of intramedullary sensory cells and promotes DRG neurogenesis.

The above findings demonstrated the role of Six1 in the transition of trunk sensory system from RB cells to DRG neurons in *Xenopus* and in DRG formation and preclusion of intramedullary sensory neuron-like cells during mouse development. Thus, *Six* genes seem to be involved in the evolutionary disappearance of intramedullary sensory neurons, such as RB cells. In *Xenopus*, Six1 expression begins in RB cells preceding their apoptotic death during the transition from RB cells to DRG (Figure 1). By comparison, the expression of SIX1 persists from the beginning of mouse DRG development (Figure 3). The expression profiles of Six1 in *Xenopus* and mouse suggest that the onset of Six1 expression during the entire developmental process of trunk sensory neurons could be different in these species, and that such difference could be the genetic basis of the disappearance of RB cells and exclusive development of DRG. However, the developmental time scale is not the same in *Xenopus* and mouse, because both species possess their own unique developmental processes acquired after the amphibian-amniotes divergence about 350 million years ago. Therefore, it is difficult to directly compare the timing of onset of Six1 expression between *Xenopus* and mouse. Accordingly, we focused on a conserved *Six1* enhancer (Six1-8) solely responsible for the expression of the gene in DRG neurons [74], and evaluated the timing of activation of mouse (mSix1-8) and *Xenopus* (xSix1-8) enhancers under the same environment. We generated transgenic *Xenopus* in which mSix1-8 or xSix1-8 drives EGFP expression to monitor enhancer activity *in vivo*[75]. In the trunk of mouse embryos, mSix1-8 activated the transcription in DRG later than E10 [74]. Surprisingly, the enhancer activity of mSix1-8 was observed in *Xenopus* RB cells at St. 25/26 (75.9 ± 3.8% RB cells, n = 5), before the endogenous expression of Six1, and at St. 41/42 (87.1 ± 1.6% RB cells, n = 12; Figure 7B). By contrast, the activity of xSix1-8 was observed only in a minor proportion of RB cells at St. 25/26 (12.8 ± 3.4% RB cells, n = 7), but became prominent at St. 41/42 (83.9 ± 2.6% RB cells, n = 12; Figure 7A), just with the appearance of endogenous expression of Six1 (Figure 1). Both enhancers showed activity in DRG at St. 49 (Figure 7A,B). These results provide strong support to our notion that Six1 expression appears earlier in trunk sensory neurons of mouse than *Xenopus* (Figure 7C). More importantly, the earlier expression is likely mediated by modification of a single enhancer that shares sequence similarity between *Xenopus* and mouse.

## Discussion

In this study, we found that Six1 is a key regulator of transition from intramedullary sensory neurons (RB cells) to extramedullary sensory neurons (DRG neurons) during *Xenopus* development (Figures 1 and 2); SIX1 precludes the appearance of intramedullary sensory neurons in mouse spinal cord by preventing NCCs migrating into the medulla (Figures 3, 4, 5 and 6); and the timing of Six1 expression in sensory neurons could be altered by changing enhancer sequences (Figure 7).

Previous studies have described the roles of Six1 in proliferation and neuronal differentiation in the sensory systems [41,42,48]. The present study describes Six1 as a key regulator of apoptosis during the process of disappearance of *Xenopus* RB cells and assigning proper location of mouse sensory neurons. Because cdk5 is involved both in apoptosis and positioning of RB cells in zebrafish [29,76], the differential functions of Six1 might be elaborated by such molecules. It is possible that the precocious disappearance of RB cells in *six1*-overexpressed *Xenopus* embryos is caused by reasons other than apoptosis. Defects in the medial migration are expected to reduce the number of RB cells incorporated in the dorsal spinal cord of *Xenopus*, analogous to mouse development in which the medial migration of NCCs leading to the emergence of intramedullary sensory neuron is precluded by *Six* genes. Identifying the mechanism of disappearance of RB cells might produce further evidence to understand the evolution of sensory neuron architecture.

Surprisingly, sensory neuron-like cells appeared in the spinal cord of *Six1/4*^*EGFP/EGFP*^, though it is unclear whether they are functional or not. What is the identity of such cells and what does their appearance imply? Intramedullary EGFP-positive cells disappeared by E12.5, probably due to apoptotic cell death (Additional file 4B), which might be induced by inappropriate environment for survival. The disappearance does not allow us to characterize the functional features of such cells as sensory neurons. The sporadic presence of such cells has been reported in amniotes, including reptiles [6] and human embryos [77,78]. Intramedullary neurons in human embryos are considered to be homologous to RB cells, because they share anatomical characteristics with amphibian RB cells [77]. Such cells are located in the region where dorsal root fibers enter the spinal cord in human, reminiscent of the position of emergence of intramedullary sensory neuron-like cells in *Six1/4*^*EGFP/EGFP*^ mice. The facts that intramedullary EGFP-positive cells in *Six1/4*^*EGFP/EGFP*^ express NTRK3, TLX3, KCNA1, RUNX1 and RUNX3; possess neurites leaving the spinal cord; and are distributed in a non-segmental manner indicate that they share certain properties with the RB cells. It has been proposed that NCCs and RB cells evolutionarily share a common origin [79]. There are a couple of molecular evidences supporting this scenario: Notch/Delta signaling is involved in segregating the two fates [24,25], Prdm1 has a role to specify both [28], and Runx1 is required for the differentiation of both RB cells and subtype of DRG neurons [64,80]. That intramedullary cells in mouse share some features with RB cells and are derived from NCCs, similar to DRG neurons, suggests common origin of intra- and extramedullary sensory neurons. Despite the fact that both DRG neurons and intramedullary EGFP-positive cells are derived from NCCs, they seem to follow segmental and non-segmental arrangement in *Six1/4*^*EGFP/EGFP*^ embryos, respectively. Segmental arrangement of DRG is determined by NCC-somite interactions [81,82]. Intramedullary EGFP-positive cells might be relieved from such signaling because they are physically isolated from the influence of somites by the basal lamina. One of the key components of NCC-somite interactions is neuropilin (NRP)/semaphorin signaling. NRP1 and NRP2 are expressed in NCCs and play essential roles in segmental formation of DRG and segmental migration of NCCs, respectively [83-85]. NRP1, but not NRP2, was expressed in intramedullary EGFP-positive cells of *Six1/4*^*EGFP/EGFP*^ (Additional file 4C), although the cells are derived from NCCs. Because genetic ablation of NRP2 results in disorganization of segmental migration of NCCs [85], non-segmental arrangement of intramedullary EGFP-positive cells in *Six1/4*^*EGFP/EGFP*^ might be due to the lack of NRP2 expression rather than the physical isolation mentioned above. Interestingly, Nrp1, but not Nrp2, is also expressed inside *Xenopus* spinal cord [86], where RB cells exist, suggesting that the combined expression of Nrps is a potential molecular basis for the non-segmental arrangement of intramedullary sensory neurons.

How do ectopic intramedullary sensory neurons appear in the *Six1/4*^*EGFP/EGFP*^ embryos? Our analysis demonstrated that the incorporation of migrating NCCs into the spinal cord is the most plausible scenario for the presence of such cells. Davidson and Keller [27] showed that frog dorsal neural tube is remodeled by medial migration and radial intercalation of lateral neural plate, including RB cells, which appear in the lateral edge of the neural plate before neural tube formation. In other words, already-specified RB cells segregate from the border and migrate medially to settle in the dorsal neural tube. A similar morphological event is observed in fish development [28,29]. The intramedullary EGFP-positive cells in *Six1/4*^*EGFP/EGFP*^ embryos show medial migration into the spinal cord from the outside. Of course, one cannot completely exclude the involvement of other mechanisms in the emergence of intramedullary EGFP-positive cells in *Six1/4*^*EGFP/EGFP*^ in addition to the medial migration of NCCs. Abnormalities in the structure that separates the CNS and PNS at the DREZ could be one such mechanism involved in the process of migration of NCCs into the spinal cord. We observed fewer BC cells and larger number of laminin gaps at the DREZ at E11.5 in *Six1/4*^*EGFP/EGFP*^ compared with *Six1/4*^*+/EGFP*^. However, the fact that intramedullary EGFP-positive cells were already present at E10.5 suggests that defects in the boundary structure is not the primary reason for the appearance of intramedullary sensory neuron-like cells in *Six1/4*^*EGFP/EGFP*^ embryos.

Our data suggest that Six1 is also involved in the evolution of extramedullary sensory neurons or the DRG system. Acquisition of new expression domains of *Six1* in the dorsal spinal cord or in the NCC lineage may have contributed to the establishment of the DRG system. The mouse *Six1* enhancer for DRG neurons exhibited earlier enhancer activity than *Xenopus six1* enhancer in RB cells and precocious overexpression of Six1 caused premature differentiation of extramedullary neurons during *Xenopus* development. This raises the possibility that the earlier expression of Six1 in the dorsal neural tube, including RB cells and NCCs, was acquired at some point during the evolution from anamniotes to amniotes. This hypothesis can be tested by analyzing the activities of the corresponding enhancers from various species including reptiles and birds.

Moreover, the directive role of Six1 in generating extramedullary sensory neurons may have deeper origins. In the amphioxus embryos, *Six1/2* seems to be expressed in type I epidermal sensory cells outside the spinal cord [87]; however, its expression has not been reported in Retzius bipolar cells, which are probably homologous to RB cells. Although there is controversy regarding the vertebrate cell type that is homologous to amphioxus extramedullary type I sensory cells [87], the expression of Six1 in extramedullary sensory neurons of both amphioxus and vertebrates is intriguing and suggests that Six1 is being recruited as one of the key regulators for generating sensory neurons outside the spinal cord, despite the different evolutionary or developmental origins of the cells.

Following the concept that altered expression of developmental regulators is an important step in morphological evolution [88], evolutionary insights have been made from experimental results obtained through the manipulation of gene expression [89,90]. Recent work has elegantly demonstrated that the acquisition of a novel enhancer results in a change in gene expression, yielding morphological diversity [91]. This suggests that the upstream mechanisms acting on the enhancer could be established prior to morphological changes. Our findings suggest that changes in the enhancer sequence that caused heterochronic shift in Six1 expression in the trunk sensory precursors of the amniote ancestors could be the genetic basis for the succession from intra- to extramedullary sensory neurons, including the disappearance of RB cells (Figure 7C).

## Conclusions

In the present study, we used inter-species gene manipulations to show the critical role of Six1 in switching of RB cells to DRG neurons in frog and in the establishment of the exclusive DRG system of mice, coupled with late or early onset of Six1 expression during sensory development. Gain- and loss-of-function of Six1 was demonstrated by experimental changes in intra- versus extramedullary sensory neurons both in frogs and mice, suggesting the conserved function of Six1 in both species despite the different sensory architectures. The species-specific activities of *Six1* enhancers, which correlate with the differential onset of Six1 expression in the trunk sensory precursors, could be the genetic basis for the different sensory architecture in frogs and mice. Our findings provide a specific example of how alterations in gene expression can contribute to substantial changes in morphology during evolution.

## Methods

### Animals

*Xenopus laevis* embryos were obtained by *in vitro* fertilization using standard methods [92] and staged according to [93]. *Six1/4*^*EGFP/EGFP*^ and *P0-Cre* mice were generated as described previously [48,57,67]. Transgenic mouse lines harboring *CAG-loxP-STOP-loxP-mRFP1* (containing CAG promoter, floxed stop sequence, *mRFP1* cDNA and rabbit β-globin polyA) or *CAG-loxP-STOP-loxP-Six1* (containing CAG promoter, floxed stop sequence, cDNA coding N-terminal flag/C-terminal myc-tagged mouse *Six1* and rabbit β-globin polyA) cassettes were generated using standard protocols and maintained by backcrossing more than 10 generations to C57BL/6. PCR was performed to verify the genotypes of offspring. Mice were housed in an environmentally controlled room. All animal experiments were carried out in a humane manner after approval of the Institutional Animal Experiment Committee of the Jichi Medical University, and in accordance with the Institutional Regulation for Animal Experiment and Fundamental Guideline for Proper Conduct of Animal Experiment and Related Activities in Academic Research Institutions under the jurisdiction of the Ministry of Education, Culture, Sports, Science and Technology of Japan.

### Immunofluorescence

Immunofluorescence was performed as described previously [61] using the following primary antibodies: guinea pig anti-SIX1 [39] (1:5,000 dilution), rat anti-SIX1 [48] (1:2,000 dilution), guinea pig anti-SIX4 [48] (1:2,000 dilution), rat anti-SIX1 (1:2,000 dilution, prepared against chick Six1), mouse anti-ISL1/2 (1:150 dilution of a mixture of hybridoma supernatants, 39.4D5 and 40.2D6, Developmental Studies Hybridoma Bank, Iowa City, USA), guinea pig anti-SOX10 (1:20,000 dilution, prepared against mouse SOX10 peptides), goat anti-NTRK3 (1:1,500 dilution, R&D Systems, Minneapolis, USA), rabbit anti-TLX3 [62] (1:10,000 dilution), rabbit anti-KCNA1 (1:2,000 dilution, Alomone Labs, Jerusalem, Israel), rabbit anti-RUNX1 (1:1,000 dilution, Abcam, Cambridge, UK), rabbit anti-laminin (1:3,000 dilution, Sigma, St. Louis, USA), rabbit anti-cleaved CASP3 (1:1,000 dilution, Cell Signaling Technology, Danvers, USA) and rabbit anti-EGR2 (1:1,000 dilution, Covance, Princeton, USA and Abcam) antibodies. To visualize immunoreactions of primary antibodies, fluorophore (Alexa Fluor 405, 488, 546, 633 and Cy5)-labeled species-specific secondary antibodies (Molecular Probes/Invitrogen, Carlsbad, USA and Amersham Biosciences, Amersham, UK) were used at 1:2,000 dilution. The immunofluorescent images were captured with FV1000 (Olympus, Tokyo, Japan) laser confocal microscope and electronically assigned to green or magenta channels.

### *In situ* hybridization

Nucleotides 355 to 907 of *X. laevis six1* (AF279254) were used to synthesize antisense RNA probes labeled with digoxigenin. *In situ* hybridization was performed as described previously [61]. Following *in situ* hybridization, Isl1/2 was detected by immunohistochemistry using mouse anti-Isl1/2 antibody (mixture of 39.4D5 and 40.2D6) and VECTASTAIN Kit (Vector Laboratories, Burlingame, USA).

### Retrograde-labeling of the neurite projection

Retrograde-labeling was performed as described previously [61] with slight modifications as follows. E11.5 mouse embryos were dissected and cultured in mouse Ringer’s solution oxygenated with carbogen (95% O_2_ plus 5% CO_2_) for 30 minutes to heal any cut edges that may incorporate the dye. Then a slit was made from the surface ectoderm overlying somites through DRG at the level of the thoracolumbar region and filled with lysine-fixable 3000 MW tetramethylrhodamine dextran (D3308, Molecular Probes/Invitrogen). The embryos were cultured for another 6 to 10 hours at room temperature to permit retrograde transport of the label and then fixed for immunofluorescence.

### GR-mediated activation of Six1 in *Xenopus* embryo

GR cDNA was amplified from pSP64T-MyoDGR [53] by PCR and inserted into pCS2+ harboring an ORF of *X. laevis six1* (AF279254) to generate a cDNA encoding C-terminal GR fusion *X. laevis* Six1 protein. The mRNA preparation, embryo manipulation and microinjection were performed as described in detail previously [94]. A total of 100 to 250 pg/embryo of β-globin or 100 to 125 pg/embryo of Six1-GR mRNA was co-injected with 400 pg/embryo of EGFP mRNA.

### siRNA-mediated knockdown of Six1 *in vivo* using electroporation

The Stealth RNAi siRNA Negative Control Med GC Duplex and Stealth siRNAs targeted to *Xenopus six1* were purchased from Invitrogen. A mixture of equimolar amounts of si1 through si3 (Figure 2M) was used for validation (Additional file 2C) and *in vivo* electroporation. *Xenopus* embryos were anesthetized with 0.075% ethyl-3-aminobenzoate methanesulfonate (Tokyo Chemical Industry, Tokyo, Japan) dissolved in 0.1× Modified Barth’s Saline (MBS) and then transferred into an electrode chamber filled with 0.1× MBS. The anode and cathode platinum electrodes (5 mm in height and 10 mm in width) were fixed on a glass dish with a 15 mm gap and integrated into a resin to establish a square chamber (CUY520P15, Nepa Gene, Ichikawa, Japan). siRNA negative control (200 to 400 μM) mixed with mRFP1 mRNA (150 ng/μl) as a control, or Six1 siRNAs (200 to 400 μM) mixed with mRFP1 mRNA or mutated Six1 mRNA (150 ng/μl) were injected into the region between the spinal cord and dorsal ectoderm in the trunk using a glass micropipette. Just after injection, the dorsoventral axis of the embryos was aligned to the current direction and then electric pulses were applied at 105 V, with 50 ms length, 950 ms interval, and 5 shocks by the electroporator, CUY-21 (Nepa Gene). Embryos were cultured in 0.1× MBS at 19°C.

### Generation of point mutations for Six1

To abolish the knockdown effect mediated by all Six1 siRNAs, three silent mutations in each siRNA-targeted regions were introduced (Figure 2M). The PCR primers were designed to contain mutations and used to amplify DNA fragments. The DNA fragments were joined using In-Fusion HD Cloning Kit (Clontech/TaKaRa, Otsu, Japan), with the protocol supplied by the manufacturer, and verified by DNA sequencing.

### Time-lapse live imaging on trunk slice culture

Live imaging analysis was performed as described previously [95] with slight modifications as follows. Transverse slices of E11 thoracolumbar region (approximately 300 μm) were prepared using Gastromaster microdissection device (GST-1, Nepa Gene). Scanning was performed with a 10× objective lens in the interval of 7.5 minutes using FV1000 (Olympus) laser confocal microscope.

### Construction of xSix1-8 and mSix1-8 plasmids

The enhancer sequences of *Xenopus* Six1-8 (JGI4.1:scaffold_68 3239865–3240552) and mouse Six1-8 (chromosome:NCBIM37: 12:74156389–74156926) were amplified from *X. tropicalis* and mouse genomes [74], respectively, by PCR and cloned into the ISpBSIISK + βGFP vector [75]. The nomenclature of enhancer sequences was based on a previous report [74].

### Generation and analysis of transgenic *Xenopus*

Transgenic *Xenopus* embryos were generated as described previously [75]. EGFP expression was detected using a rabbit anti-EGFP antibody (1:2,000 dilution, MBL, Nagoya, Japan).

### Statistical analysis

All values are expressed as mean ± standard error of the mean of at least three independent assays or littermates. Differences from the control experiments were evaluated with Student’s t-test. A probability of less than 5% was considered statistically significant.

## Abbreviations

BC: boundary cap; CNS: central nervous system; Dex: dexamethasone; DREZ: dorsal root entry zone; DRG: dorsal root ganglia; E: embryonic day; EGFP: enhanced green fluorescent protein; GR: glucocorticoid receptor ligand-binding domain; MEP: motor exit point; mRFP: monomeric red fluorescent protein; MBS: Modified Barth’s saline; NCC: neural crest cell; ORF: open reading frame; PCR: polymerase chain reaction; PNS: peripheral nervous system; RB: Rohon-Beard; siRNA: small interfering RNA; St: Stage.

## Competing interests

The authors declare that they have no competing interests.

## Authors’ contributions

HY was involved in the conception and design, data collection, analysis and interpretation, and manuscript writing; MS, HOc, HOg and NU in data collection and data analysis; KI and KY in provision of study material; SS in data interpretation and provision of study material; KK in conception and design, data analysis and interpretation, and manuscript writing. All authors read and approved the final manuscript.

## Supplementary Material

Additional file 1 **Absence of *six *** **gene expression in the dorsal neural tube of *Xenopus *** **embryo during early development. (A,B)***six1*, **(C,D)***six4* (AF276994) and **(E,F)***six4* (AF276995) mRNAs are not detected by whole-mount *in situ* hybridization (purple staining) in the dorsal neural tube of *Xenopus* embryos at St. 16/17 **(A,C,E)** and 25/26 **(B,D,F)**. Left: rostral side. Scale bars: 0.5 mm. Click here for file

Additional file 2 **Gain- and loss-of-function of Six1 affects primary sensory development. (A)** Schematic representation of GR-mediated activation of Six1. **(B)** Immunofluorescence of Tlx3 and Kcna1 in *Xenopus* primary sensory neurons. The nuclei and cytoplasm of *Xenopus* RB cells (arrowheads) are labeled with anti-Isl1/2 (green) and HNK-1 (magenta), respectively. Tlx3 (green) and Kcna1 (green) are also detected in the nuclei and the cell membrane/cytoplasm of these cells, respectively. DRG neurons are positive for both Isl1/2 (green) and Tlx3 (magenta). Scale bars: 25 μm. **(C)** Verification of the knockdown efficacy of a mixture of Six1 siRNAs and the resistance of mutated Six1 to Six1 siRNAs. Plasmids containing the FLAG-tagged Six1 (pCS2-FLAG-Six1) or the mutated Six1 (pCS2-FLAG-Six1-silent, Figure 2M) are transfected into HEK293 cell line with negative control siRNA (nega.ctrl.si) or a mixture of Six1 siRNAs (Six1 siRNAs). The expression plasmid for EGFP (pEGFP) is co-transfected to monitor the efficiency of transfection. Protein levels are determined by western blotting using anti-FLAG and anti-EGFP antibodies. The signal intensity is analyzed densitometrically and displayed in bar graph, normalized to EGFP level and expressed relative to that of negative control siRNA. Note that Six1 siRNAs show efficient protein knockdown, which is abolished by mutations in the siRNA target sequences. **(D)** Reduction of RB cell number in the trunk region. *Xenopus* development is associated with a fall in the total number of RB cells located in the entire spinal cord, starting at St. 46 [9]. To analyze the phenotypes in electropolated area, the number of RB cells in the spinal cord at the level of somites 1 through 9 (between two red lines) is re-evaluated and displayed in bar graph (n = 5 for each stage, data are mean ± standard error of the mean). Cell numbers started to decrease earlier than that of whole spinal cord. Scale bar: 1 mm. Click here for file

Additional file 3 **Both SIX1 and SIX4 are expressed in DRG. (A)** Specificities of anti-SIX1 and anti-SIX4 antibodies are validated by using *Six1* or *Six4* single homozygous knockout mice, *Six1*^*-/-*^ or *Six4*^*-/-*^. To evaluate the specificities of the antibodies, the trigeminal ganglia (Vg) are subjected to immunofluorescence staining, in which both SIX1 and SIX4 are expressed during the development [48]. The rat polyclonal antibody against mouse SIX1 [48] detects SIX1 protein in *Six4*^*-/-*^ embryo (denoted as *Six4*^*LacZ/LacZ*^) [50], but not in *Six1*^*-/-*^ embryo (denoted as *Six1*^*EGFP/EGFP*^) [41]. The guinea pig polyclonal antibody against mouse SIX4 [39] recognizes SIX4 in *Six1*^*EGFP/EGFP*^ embryo, but none in *Six4*^*LacZ/LacZ*^. These results show lack of cross-reactivity of anti-SIX1 and anti-SIX4 antibodies with SIX4 and SIX1, respectively. Bottom line shows merged images; SIX1 and SIX4 in wild type, EGFP expressed from *Six1* knockout alleles and SIX4 in *Six1*^*EGFP/EGFP*^, SIX1 and β-galactosidase expressed from *Six4* knockout alleles in *Six4*^*LacZ/LacZ*^. Scale bars: 50 μm. **(B)** Similar distribution of SIX1 and SIX4 in developing mouse DRG. In E11.5 mouse embryo, the majority of SIX1-positive-cells in DRG (green) are labeled with SIX4 immunofluorescence (magenta), as shown in the merged panel. The relative intensities of immunofluorescent signals for SIX1 and SIX4 vary among DRG neurons. Dashed lines demarcate the position of the ectoderm and spinal cord (sc). Scale bar: 50 μm. Click here for file

Additional file 4 **Intramedullary EGFP-positive cells in *Six1/4***^***EGFP/EGFP ***^**mice are positive for RUNX1, RUNX3 cleaved CASP3 and NRP1. (A)** The majority of intramedullary EGFP-positive cells in *Six1/4*^*EGFP/EGFP*^ embryos (green in spinal cord (sc)) are positive for RUNX1 (magenta) and RUNX3 (magenta), as pointed out with arrowheads. Top line shows embryonic days of embryos. RUNX3 is detected at E11, one day earlier than that of RUNX1. **(B)** A substantial number of cleaved CASP3 (cCasp3)-positive cells (magenta) are observed in the spinal cords of *Six1/4*^*EGFP/EGFP*^ at E12 and these cells are also positive for EGFP (green, arrowheads), whereas no such cells are observed in the *Six1/4*^*+/EGFP*^ spinal cord. **(C)** The majority of intramedullary EGFP-positive cells in *Six1/4*^*EGFP/EGFP*^ embryos (green in sc) are positive for NRP1 (magenta), but not for NRP2 (magenta), as indicated by arrowheads. Dashed lines demarcate the position of the ectoderm and sc. Scale bars: 50 μm. Click here for file

Additional file 5 **Medial migration of EGFP-positive cells of *Six1/4***^***EGFP/EGFP***^. Time-lapse confocal microscopy images of EGFP-positive cells on slice culture of E11 *Six1/4*^*EGFP/EGFP*^ lumbar region were acquired every 7.5 minutes for 8 hours and animated in 60 ms/frame. Snapshots at the indicated time points are shown in Figure 6I. The animated sequence that harbors the dotted line demarcating the edge of the spinal cord and magenta-filled square and triangle pointing to EGFP-positive cells follows the one without any labels. Click here for file

Additional file 6 **Number but not length of individual laminin gaps is increased in *Six1/4***^***EGFP/EGFP ***^**DREZ. (A)** Number and **(B)** length of individual laminin gaps in 50 μm length of basal lamina covering the primordium of the dorsal funiculus. For all measurements, at least five sections from three embryos were used per genotype. Data are mean ± standard error of the mean. *p <0.005. Click here for file

## References

[B1] Beard J On the early development of *Lepidosteus osseus* - preliminary notice Proc R Soc Lond 1889 12 108 118

[B2] Bernhardt RR Chitnis AB Lindamer L Kuwada JY Identification of spinal neurons in the embryonic and larval zebrafish J Comp Neurol 1990 12 603 616 1702120 10.1002/cne.903020315

[B3] Coghill GE Correlated anatomical and physiological studies on the growth of the nervous system of Amphibia. 1. The afferent system of the trunk of Amblystoma J Comp Neurol 1914 12 161 233

[B4] Hughes A The development of the primary sensory system in *Xenopus laevis* (Daudin) J Anat 1957 12 323 338 13448990 PMC1244917

[B5] Eccles JC Schadé JP Organization of the Spinal Cord 1964 Amsterdam: Elsevier

[B6] Ariëns Kappers CU Huber GC Crosby EC The Comparative Anatomy of the Nervous System of Vertebrates, Including Man 1967 New York: Hafner (Originally published in 1936)

[B7] Fritzsch B Northcutt RG Cranial and spinal nerve organization in amphioxus and lampreys: evidence for an ancestral craniate pattern Acta Anat (Basel) 1993 12 96 109 8109201 10.1159/000147529

[B8] Hartenstein V Early pattern of neuronal differentiation in the *Xenopus* embryonic brainstem and spinal cord J Comp Neurol 1993 12 213 231 8423241 10.1002/cne.903280205

[B9] Lamborghini JE Disappearance of Rohon-Beard neurons from the spinal cord of larval *Xenopus laevis* J Comp Neurol 1987 12 47 55 3680623 10.1002/cne.902640105

[B10] Bone Q The central nervous system in larval acraniates Q J Microsc Sci 1959 12 509 527

[B11] Lacalli TC Kelly SJ Sensory pathways in amphioxus larvae II. Dorsal tracts and translumenal cells Acta Zool-Stockholm 2003 12 1 13

[B12] Bone Q The central nervous system in amphioxus J Comp Neurol 1960 12 27 64

[B13] Johnston JB The cranial and spinal ganglia and the viscero-motor roots in amphioxus Biol Bull 1905 12 112 127

[B14] Whiting HP Nervous structure of the spinal cord of the young larval brook-lamprey Q J Microsc Sci 1948 12 359 383 18109176

[B15] Nakao T Ishizawa A Development of the spinal nerves in the lamprey: I. Rohon-Beard cells and interneurons J Comp Neurol 1987 12 342 355 3571509 10.1002/cne.902560304

[B16] Beard J The transient ganglion cells and their nerves in Raja batis Anat Anzeiger 1892 12 191 206

[B17] Kuratani S Horigome N Developmental morphology of branchiomeric nerves in a cat shark, *Scyliorhinus torazame*, with special reference to rhombomeres, cephalic mesoderm, and distribution patterns of cephalic crest cells Zool Sci 2000 12 893 909

[B18] Kuwada JY Bernhardt RR Nguyen N Development of spinal neurons and tracts in the zebrafish embryo J Comp Neurol 1990 12 617 628 2262604 10.1002/cne.903020316

[B19] Eichler VB Porter RA Rohon-Beard cells in frog development: a study of temporal and spatial changes in a transient cell population J Comp Neurol 1981 12 121 130 6975782 10.1002/cne.902030110

[B20] Forehand CJ Farel PB Spinal cord development in anuran larvae: I. Primary and secondary neurons J Comp Neurol 1982 12 386 394 6982287 10.1002/cne.902090408

[B21] Kollros JJ Bovbjerg AM Growth and death of Rohon-Beard cells in *Rana pipiens* and *Ceratophrys ornata* J Morphol 1997 12 67 78 9068202 10.1002/(SICI)1097-4687(199704)232:1<67::AID-JMOR4>3.0.CO;2-L

[B22] Schlosser G Roth G Evolution of nerve development in frogs. II. Modified development of the peripheral nervous system in the direct-developing frog *Eleutherodactylus coqui* (Leptodactylidae) Brain Behav Evol 1997 12 94 128 9261555 10.1159/000113325

[B23] Rossi CC Hernandez-Lagunas L Zhang C Choi IF Kwok L Klymkowsky M Artinger KB Rohon-Beard sensory neurons are induced by BMP4 expressing non-neural ectoderm in *Xenopus laevis* Dev Biol 2008 12 351 361 18191829 10.1016/j.ydbio.2007.11.036 PMC2262044

[B24] Cornell RA Eisen JS Delta signaling mediates segregation of neural crest and spinal sensory neurons from zebrafish lateral neural plate Development 2000 12 2873 2882 10851132 10.1242/dev.127.13.2873

[B25] Cornell RA Eisen JS Delta/Notch signaling promotes formation of zebrafish neural crest by repressing Neurogenin 1 function Development 2002 12 2639 2648 12015292 10.1242/dev.129.11.2639

[B26] Theveneau E Mayor R Neural crest delamination and migration: from epithelium-to-mesenchyme transition to collective cell migration Dev Biol 2012 12 34 54 22261150 10.1016/j.ydbio.2011.12.041

[B27] Davidson LA Keller RE Neural tube closure in *Xenopus laevis* involves medial migration, directed protrusive activity, cell intercalation and convergent extension Development 1999 12 4547 4556 10498689 10.1242/dev.126.20.4547

[B28] Rossi CC Kaji T Artinger KB Transcriptional control of Rohon-Beard sensory neuron development at the neural plate border Dev Dyn 2009 12 931 943 19301392 10.1002/dvdy.21915 PMC2755227

[B29] Tanaka H Morimura R Ohshima T Dpysl2 (CRMP2) and Dpysl3 (CRMP4) phosphorylation by Cdk5 and DYRK2 is required for proper positioning of Rohon-Beard neurons and neural crest cells during neurulation in zebrafish Dev Biol 2012 12 223 236 22898304 10.1016/j.ydbio.2012.07.032

[B30] Metcalfe WK Myers PZ Trevarrow B Bass MB Kimmel CB Primary neurons that express the L2/HNK-1 carbohydrate during early development in the zebrafish Development 1990 12 491 504 1723944 10.1242/dev.110.2.491

[B31] Ribera AB Nusslein-Volhard C Zebrafish touch-insensitive mutants reveal an essential role for the developmental regulation of sodium current J Neurosci 1998 12 9181 9191 9801358 10.1523/JNEUROSCI.18-22-09181.1998 PMC6792885

[B32] Williams JA Barrios A Gatchalian C Rubin L Wilson SW Holder N Programmed cell death in zebrafish rohon beard neurons is influenced by TrkC1/NT-3 signaling Dev Biol 2000 12 220 230 11023682 10.1006/dbio.2000.9860

[B33] Klymkowsky MW Rossi CC Artinger KB Mechanisms driving neural crest induction and migration in the zebrafish and *Xenopus laevis* Cell Adh Migr 2010 12 595 608 20962584 10.4161/cam.4.4.12962 PMC3011258

[B34] Bricaud O Collazo A The transcription factor six1 inhibits neuronal and promotes hair cell fate in the developing zebrafish (*Danio rerio*) inner ear J Neurosci 2006 12 10438 10451 17035528 10.1523/JNEUROSCI.1025-06.2006 PMC6674689

[B35] Bricaud O Collazo A Balancing cell numbers during organogenesis: Six1a differentially affects neurons and sensory hair cells in the inner ear Dev Biol 2011 12 191 201 21745464 10.1016/j.ydbio.2011.06.035 PMC3156292

[B36] Schlosser G Awtry T Brugmann SA Jensen ED Neilson K Ruan G Stammler A Voelker D Yan B Zhang C Klymkowsky MW Moody SA Eya1 and Six1 promote neurogenesis in the cranial placodes in a SoxB1-dependent fashion Dev Biol 2008 12 199 214 18571637 10.1016/j.ydbio.2008.05.523 PMC2671077

[B37] Brugmann SA Pandur PD Kenyon KL Pignoni F Moody SA Six1 promotes a placodal fate within the lateral neurogenic ectoderm by functioning as both a transcriptional activator and repressor Development 2004 12 5871 5881 15525662 10.1242/dev.01516

[B38] Ikeda K Kageyama R Suzuki Y Kawakami K Six1 is indispensable for production of functional progenitor cells during olfactory epithelial development Int J Dev Biol 2010 12 1453 1464 21302255 10.1387/ijdb.093041ki

[B39] Ikeda K Ookawara S Sato S Ando Z Kageyama R Kawakami K Six1 is essential for early neurogenesis in the development of olfactory epithelium Dev Biol 2007 12 53 68 17880938 10.1016/j.ydbio.2007.08.020

[B40] Laclef C Souil E Demignon J Maire P Thymus, kidney and craniofacial abnormalities in Six 1 deficient mice Mech Dev 2003 12 669 679 12834866 10.1016/s0925-4773(03)00065-0

[B41] Ozaki H Nakamura K Funahashi J Ikeda K Yamada G Tokano H Okamura HO Kitamura K Muto S Kotaki H Sudo K Horai R Iwakura Y Kawakami K Six1 controls patterning of the mouse otic vesicle Development 2004 12 551 562 14695375 10.1242/dev.00943

[B42] Zheng W Huang L Wei ZB Silvius D Tang B Xu PX The role of Six1 in mammalian auditory system development Development 2003 12 3989 4000 12874121 10.1242/dev.00628 PMC3873880

[B43] Zou D Silvius D Fritzsch B Xu PX Eya1 and Six1 are essential for early steps of sensory neurogenesis in mammalian cranial placodes Development 2004 12 5561 5572 15496442 10.1242/dev.01437 PMC3882150

[B44] Ito T Noguchi Y Yashima T Kitamura K SIX1 mutation associated with enlargement of the vestibular aqueduct in a patient with branchio-oto syndrome Laryngoscope 2006 12 796 799 16652090 10.1097/01.mlg.0000209096.40400.96

[B45] Ruf RG Xu PX Silvius D Otto EA Beekmann F Muerb UT Kumar S Neuhaus TJ Kemper MJ Raymond RM Jr Brophy PD Berkman J Gattas M Hyland V Ruf EM Schwartz C Chang EH Smith RJ Stratakis CA Weil D Petit C Hildebrandt F SIX1 mutations cause branchio-oto-renal syndrome by disruption of EYA1-SIX1-DNA complexes Proc Natl Acad Sci U S A 2004 12 8090 8095 15141091 10.1073/pnas.0308475101 PMC419562

[B46] Kochhar A Orten DJ Sorensen JL Fischer SM Cremers CW Kimberling WJ Smith RJ SIX1 mutation screening in 247 branchio-oto-renal syndrome families: a recurrent missense mutation associated with BOR Hum Mutat 2008 12 565 18330911 10.1002/humu.20714

[B47] Kobayashi H Kawakami K Asashima M Nishinakamura R Six1 and Six4 are essential for Gdnf expression in the metanephric mesenchyme and ureteric bud formation, while Six1 deficiency alone causes mesonephric-tubule defects Mech Dev 2007 12 290 303 17300925 10.1016/j.mod.2007.01.002

[B48] Konishi Y Ikeda K Iwakura Y Kawakami K Six1 and Six4 promote survival of sensory neurons during early trigeminal gangliogenesis Brain Res 2006 12 93 102 16938278 10.1016/j.brainres.2006.07.103

[B49] Zou D Silvius D Davenport J Grifone R Maire P Xu PX Patterning of the third pharyngeal pouch into thymus/parathyroid by Six and Eya1 Dev Biol 2006 12 499 512 16530750 10.1016/j.ydbio.2005.12.015 PMC3882147

[B50] Ozaki H Watanabe Y Takahashi K Kitamura K Tanaka A Urase K Momoi T Sudo K Sakagami J Asano M Iwakura Y Kawakami K Six4, a putative myogenin gene regulator, is not essential for mouse embryonal development Mol Cell Biol 2001 12 3343 3350 11313460 10.1128/MCB.21.10.3343-3350.2001 PMC100256

[B51] Korzh V Edlund T Thor S Zebrafish primary neurons initiate expression of the LIM homeodomain protein Isl-1 at the end of gastrulation Development 1993 12 417 425 8223269 10.1242/dev.118.2.417

[B52] Inoue A Takahashi M Hatta K Hotta Y Okamoto H Developmental regulation of islet-1 mRNA expression during neuronal differentiation in embryonic zebrafish Dev Dyn 1994 12 1 11 8167375 10.1002/aja.1001990102

[B53] Kolm PJ Sive HL Efficient hormone-inducible protein function in *Xenopus laevis* Dev Biol 1995 12 267 272 7556904 10.1006/dbio.1995.1279

[B54] Jacobson M Rohon-Beard neuron origin from blastomeres of the 16-cell frog embryo J Neurosci 1981 12 918 922 7346595 10.1523/JNEUROSCI.01-08-00918.1981 PMC6564228

[B55] Lamborghini JE Rohon-beard cells and other large neurons in *Xenopus* embryos originate during gastrulation J Comp Neurol 1980 12 323 333 7364967 10.1002/cne.901890208

[B56] Patterson KD Krieg PA Hox11-family genes XHox11 and XHox11L2 in *Xenopus*: XHox11L2 expression is restricted to a subset of the primary sensory neurons Dev Dyn 1999 12 34 43 9915574 10.1002/(SICI)1097-0177(199901)214:1<34::AID-DVDY4>3.0.CO;2-R

[B57] Suzuki Y Ikeda K Kawakami K Expression of Six1 and Six4 in mouse taste buds J Mol Histol 2010 12 205 214 20668922 10.1007/s10735-010-9280-8

[B58] Ando Z Sato S Ikeda K Kawakami K Slc12a2 is a direct target of two closely related homeobox proteins, Six1 and Six4 FEBS J 2005 12 3026 3041 15955062 10.1111/j.1742-4658.2005.04716.x

[B59] Ozaki S Snider WD Initial trajectories of sensory axons toward laminar targets in the developing mouse spinal cord J Comp Neurol 1997 12 215 229 9100133

[B60] Mu X Silos-Santiago I Carroll SL Snider WD Neurotrophin receptor genes are expressed in distinct patterns in developing dorsal root ganglia J Neurosci 1993 12 4029 4041 8366358 10.1523/JNEUROSCI.13-09-04029.1993 PMC6576438

[B61] Sharma K Sheng HZ Lettieri K Li H Karavanov A Potter S Westphal H Pfaff SL LIM homeodomain factors Lhx3 and Lhx4 assign subtype identities for motor neurons Cell 1998 12 817 828 9865699 10.1016/s0092-8674(00)81704-3

[B62] Muller T Anlag K Wildner H Britsch S Treier M Birchmeier C The bHLH factor Olig3 coordinates the specification of dorsal neurons in the spinal cord Genes Dev 2005 12 733 743 15769945 10.1101/gad.326105 PMC1065726

[B63] Ribera AB Nguyen DA Primary sensory neurons express a Shaker-like potassium channel gene J Neurosci 1993 12 4988 4996 8229210 10.1523/JNEUROSCI.13-11-04988.1993 PMC6576330

[B64] Park BY Hong CS Weaver JR Rosocha EM Saint-Jeannet JP Xaml1/Runx1 is required for the specification of Rohon-Beard sensory neurons in *Xenopus* Dev Biol 2012 12 65 75 22173066 10.1016/j.ydbio.2011.11.016 PMC3257842

[B65] Park BY Saint-Jeannet JP Expression analysis of Runx3 and other Runx family members during *Xenopus* development Gene Expr Patterns 2010 12 159 166 20433948 10.1016/j.gep.2010.04.004 PMC2896033

[B66] Heathcote RD Chen A A nonrandom interneuronal pattern in the developing frog spinal cord J Comp Neurol 1993 12 437 448 8095054 10.1002/cne.903280309

[B67] Yamauchi Y Abe K Mantani A Hitoshi Y Suzuki M Osuzu F Kuratani S Yamamura K A novel transgenic technique that allows specific marking of the neural crest cell lineage in mice Dev Biol 1999 12 191 203 10419695 10.1006/dbio.1999.9323

[B68] Niederlander C Lumsden A Late emigrating neural crest cells migrate specifically to the exit points of cranial branchiomotor nerves Development 1996 12 2367 2374 8756282 10.1242/dev.122.8.2367

[B69] Vermeren M Maro GS Bron R McGonnell IM Charnay P Topilko P Cohen J Integrity of developing spinal motor columns is regulated by neural crest derivatives at motor exit points Neuron 2003 12 403 415 12575949 10.1016/s0896-6273(02)01188-1

[B70] Maro GS Vermeren M Voiculescu O Melton L Cohen J Charnay P Topilko P Neural crest boundary cap cells constitute a source of neuronal and glial cells of the PNS Nat Neurosci 2004 12 930 938 15322547 10.1038/nn1299

[B71] Coulpier F Decker L Funalot B Vallat JM Garcia-Bragado F Charnay P Topilko P CNS/PNS boundary transgression by central glia in the absence of Schwann cells or Krox20/Egr2 function J Neurosci 2010 12 5958 5967 20427655 10.1523/JNEUROSCI.0017-10.2010 PMC6632613

[B72] Schneider-Maunoury S Topilko P Seitandou T Levi G Cohen-Tannoudji M Pournin S Babinet C Charnay P Disruption of Krox-20 results in alteration of rhombomeres 3 and 5 in the developing hindbrain Cell 1993 12 1199 1214 7903221 10.1016/0092-8674(93)90329-o

[B73] Golding JP Cohen J Border controls at the mammalian spinal cord: late-surviving neural crest boundary cap cells at dorsal root entry sites may regulate sensory afferent ingrowth and entry zone morphogenesis Mol Cell Neurosci 1997 12 381 396 9361276 10.1006/mcne.1997.0647

[B74] Sato S Ikeda K Shioi G Nakao K Yajima H Kawakami K Regulation of Six1 expression by evolutionarily conserved enhancers in tetrapods Dev Biol 2012 12 95 108 22659139 10.1016/j.ydbio.2012.05.023

[B75] Ogino H Fisher M Grainger RM Convergence of a head-field selector Otx2 and Notch signaling: a mechanism for lens specification Development 2008 12 249 258 18057103 10.1242/dev.009548 PMC3918164

[B76] Kanungo J Li BS Zheng Y Pant HC Cyclin-dependent kinase 5 influences Rohon-Beard neuron survival in zebrafish J Neurochem 2006 12 251 259 16911583 10.1111/j.1471-4159.2006.04114.x PMC5998666

[B77] Humphrey T Primitive neurons in the embryonic human central nervous system J Comp Neurol 1944 12 1 45

[B78] Youngstrom KA Intramedullary sensory type ganglion cells in the spinal cord of human embryos J Comp Neurol 1944 12 47 53

[B79] Donoghue PC Graham A Kelsh RN The origin and evolution of the neural crest Bioessays 2008 12 530 541 18478530 10.1002/bies.20767 PMC2692079

[B80] Chen CL Broom DC Liu Y de Nooij JC Li Z Cen C Samad OA Jessell TM Woolf CJ Ma Q Runx1 determines nociceptive sensory neuron phenotype and is required for thermal and neuropathic pain Neuron 2006 12 365 377 16446141 10.1016/j.neuron.2005.10.036

[B81] Gammill LS Roffers-Agarwal J Division of labor during trunk neural crest development Dev Biol 2010 12 555 565 20399766 10.1016/j.ydbio.2010.04.009 PMC2914176

[B82] Kuriyama S Mayor R Molecular analysis of neural crest migration Philos Trans R Soc Lond B Biol Sci 2008 12 1349 1362 18198151 10.1098/rstb.2007.2252 PMC2610123

[B83] Kawasaki T Bekku Y Suto F Kitsukawa T Taniguchi M Nagatsu I Nagatsu T Itoh K Yagi T Fujisawa H Requirement of neuropilin 1-mediated Sema3A signals in patterning of the sympathetic nervous system Development 2002 12 671 680 11830568 10.1242/dev.129.3.671

[B84] Gammill LS Gonzalez C Gu C Bronner-Fraser M Guidance of trunk neural crest migration requires neuropilin 2/semaphorin 3F signaling Development 2006 12 99 106 16319111 10.1242/dev.02187

[B85] Roffers-Agarwal J Gammill LS Neuropilin receptors guide distinct phases of sensory and motor neuronal segmentation Development 2009 12 1879 1888 19403658 10.1242/dev.032920 PMC2730397

[B86] Koestner U Shnitsar I Linnemannstons K Hufton AL Borchers A Semaphorin and neuropilin expression during early morphogenesis of *Xenopus laevis* Dev Dyn 2008 12 3853 3863 18985750 10.1002/dvdy.21785

[B87] Kozmik Z Holland ND Kreslova J Oliveri D Schubert M Jonasova K Holland LZ Pestarino M Benes V Candiani S Pax-Six-Eya-Dach network during amphioxus development: conservation *in vitro* but context specificity *in vivo* Dev Biol 2007 12 143 159 17477914 10.1016/j.ydbio.2007.03.009

[B88] Carroll SB Evo-devo and an expanding evolutionary synthesis: a genetic theory of morphological evolution Cell 2008 12 25 36 18614008 10.1016/j.cell.2008.06.030

[B89] Koshiba-Takeuchi K Mori AD Kaynak BL Cebra-Thomas J Sukonnik T Georges RO Latham S Beck L Henkelman RM Black BL Olson EN Wade J Takeuchi JK Nemer M Gilbert SF Bruneau BG Reptilian heart development and the molecular basis of cardiac chamber evolution Nature 2009 12 95 98 19727199 10.1038/nature08324 PMC2753965

[B90] Shim S Kwan KY Li M Lefebvre V Sestan N Cis-regulatory control of corticospinal system development and evolution Nature 2012 12 74 79 22678282 10.1038/nature11094 PMC3375921

[B91] Freitas R Gomez-Marin C Wilson JM Casares F Gomez-Skarmeta JL Hoxd13 contribution to the evolution of vertebrate appendages Dev Cell 2012 12 1219 1229 23237954 10.1016/j.devcel.2012.10.015

[B92] Sive HL Grainger RM Harland RM Early Development of Xenopus laevis 2000 New York: Cold Spring Harbor Laboratory Press

[B93] Nieuwkoop PD Faber J Normal Table of Xenopus laevis (Daudin) 1967 Amsterdam: North-Holland Publishing Company

[B94] Suzuki M Hara Y Takagi C Yamamoto TS Ueno N MID1 and MID2 are required for *Xenopus* neural tube closure through the regulation of microtubule organization Development 2010 12 2329 2339 20534674 10.1242/dev.048769

[B95] Takahashi M Osumi N Live Imaging of neuroepithelial cells in the rat spinal cord by confocal laser-scanning microscopy Bionanotechnology Based Future Medical Engineering Proceedings of the Final Symposium of the Tohoku University 21st Century Center of Excellence Program 2006 London: Imperial College Press 211 220

