# Supplementary figures and images for: Six1 is a key regulator of the developmental and evolutionary architecture of sensory neurons in craniates
Source: BMC Biol. 2014 May 29;12:40. doi: 10.1186/1741-7007-12-40 (PMC4084797; doi:10.1186/1741-7007-12-40)

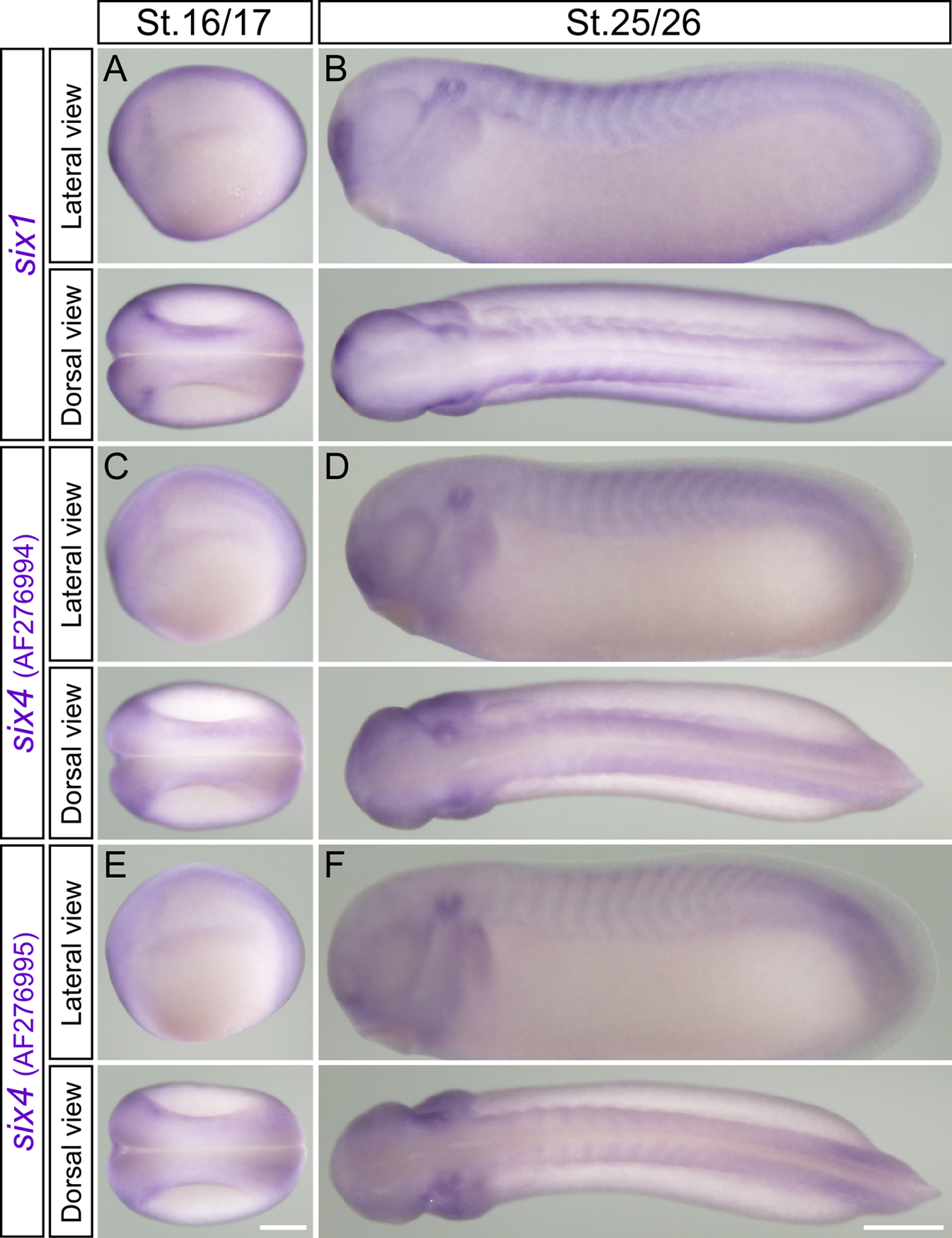

Supplement: Additional file 1 — Absence of six gene expression in the dorsal neural tube of Xenopus embryo during early development. (A,B)six1, (C,D)six4 (AF276994) and (E,F)six4 (AF276995) mRNAs are not detected by whole-mount in situ hybridization (purple staining) in the dorsal neural tube of Xenopus embryos at St. 16/17 (A,C,E) and 25/26 (B,D,F). Left: rostral side. Scale bars: 0.5 mm. [file 1741-7007-12-40-S1.tiff]

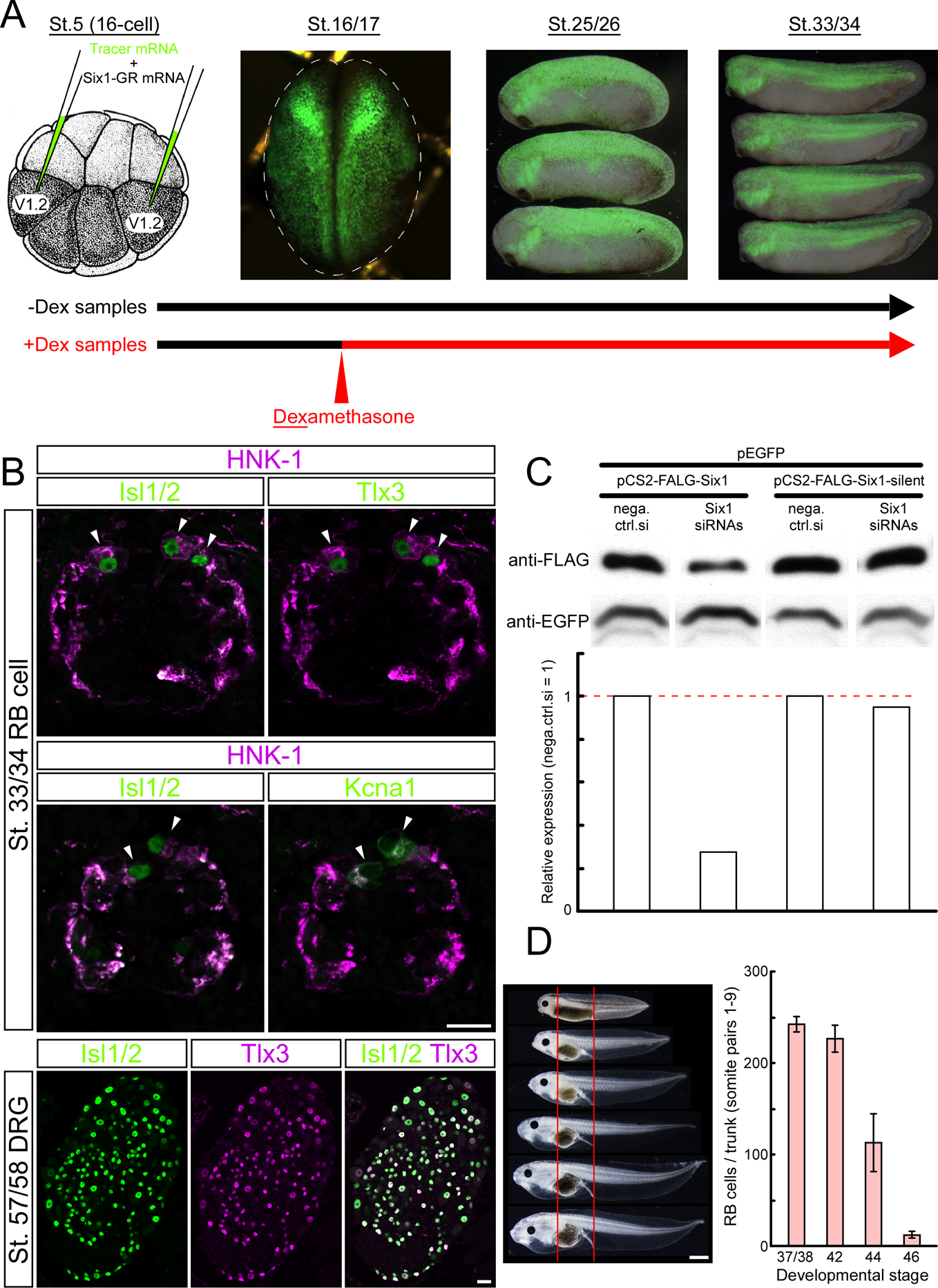

Supplement: Additional file 2 — Gain- and loss-of-function of Six1 affects primary sensory development. (A) Schematic representation of GR-mediated activation of Six1. (B) Immunofluorescence of Tlx3 and Kcna1 in Xenopus primary sensory neurons. The nuclei and cytoplasm of Xenopus RB cells (arrowheads) are labeled with anti-Isl1/2 (green) and HNK-1 (magenta), respectively. Tlx3 (green) and Kcna1 (green) are also detected in the nuclei and the cell membrane/cytoplasm of these cells, respectively. DRG neurons are positive for both Isl1/2 (green) and Tlx3 (magenta). Scale bars: 25 μm. (C) Verification of the knockdown efficacy of a mixture of Six1 siRNAs and the resistance of mutated Six1 to Six1 siRNAs. Plasmids containing the FLAG-tagged Six1 (pCS2-FLAG-Six1) or the mutated Six1 (pCS2-FLAG-Six1-silent, Figure 2M) are transfected into HEK293 cell line with negative control siRNA (nega.ctrl.si) or a mixture of Six1 siRNAs (Six1 siRNAs). The expression plasmid for EGFP (pEGFP) is co-transfected to monitor the efficiency of transfection. Protein levels are determined by western blotting using anti-FLAG and anti-EGFP antibodies. The signal intensity is analyzed densitometrically and displayed in bar graph, normalized to EGFP level and expressed relative to that of negative control siRNA. Note that Six1 siRNAs show efficient protein knockdown, which is abolished by mutations in the siRNA target sequences. (D) Reduction of RB cell number in the trunk region. Xenopus development is associated with a fall in the total number of RB cells located in the entire spinal cord, starting at St. 46 [9]. To analyze the phenotypes in electropolated area, the number of RB cells in the spinal cord at the level of somites 1 through 9 (between two red lines) is re-evaluated and displayed in bar graph (n = 5 for each stage, data are mean ± standard error of the mean). Cell numbers started to decrease earlier than that of whole spinal cord. Scale bar: 1 mm. [file 1741-7007-12-40-S2.tiff]

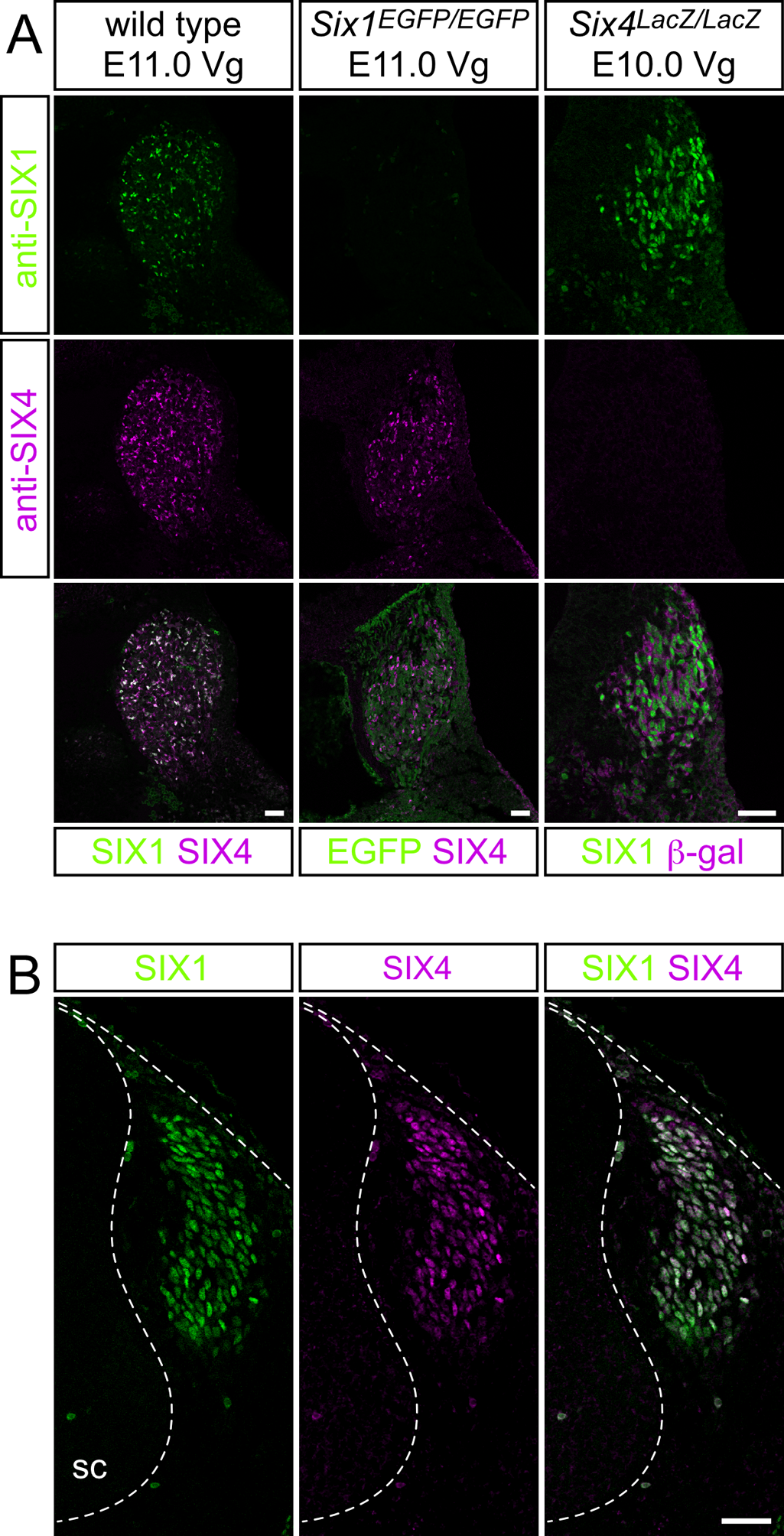

Supplement: Additional file 3 — Both SIX1 and SIX4 are expressed in DRG. (A) Specificities of anti-SIX1 and anti-SIX4 antibodies are validated by using Six1 or Six4 single homozygous knockout mice, Six1 -/- or Six4 -/- . To evaluate the specificities of the antibodies, the trigeminal ganglia (Vg) are subjected to immunofluorescence staining, in which both SIX1 and SIX4 are expressed during the development [48]. The rat polyclonal antibody against mouse SIX1 [48] detects SIX1 protein in Six4 -/- embryo (denoted as Six4 LacZ/LacZ ) [50], but not in Six1 -/- embryo (denoted as Six1 EGFP/EGFP ) [41]. The guinea pig polyclonal antibody against mouse SIX4 [39] recognizes SIX4 in Six1 EGFP/EGFP embryo, but none in Six4 LacZ/LacZ . These results show lack of cross-reactivity of anti-SIX1 and anti-SIX4 antibodies with SIX4 and SIX1, respectively. Bottom line shows merged images; SIX1 and SIX4 in wild type, EGFP expressed from Six1 knockout alleles and SIX4 in Six1 EGFP/EGFP , SIX1 and β-galactosidase expressed from Six4 knockout alleles in Six4 LacZ/LacZ . Scale bars: 50 μm. (B) Similar distribution of SIX1 and SIX4 in developing mouse DRG. In E11.5 mouse embryo, the majority of SIX1-positive-cells in DRG (green) are labeled with SIX4 immunofluorescence (magenta), as shown in the merged panel. The relative intensities of immunofluorescent signals for SIX1 and SIX4 vary among DRG neurons. Dashed lines demarcate the position of the ectoderm and spinal cord (sc). Scale bar: 50 μm. [file 1741-7007-12-40-S3.tiff]

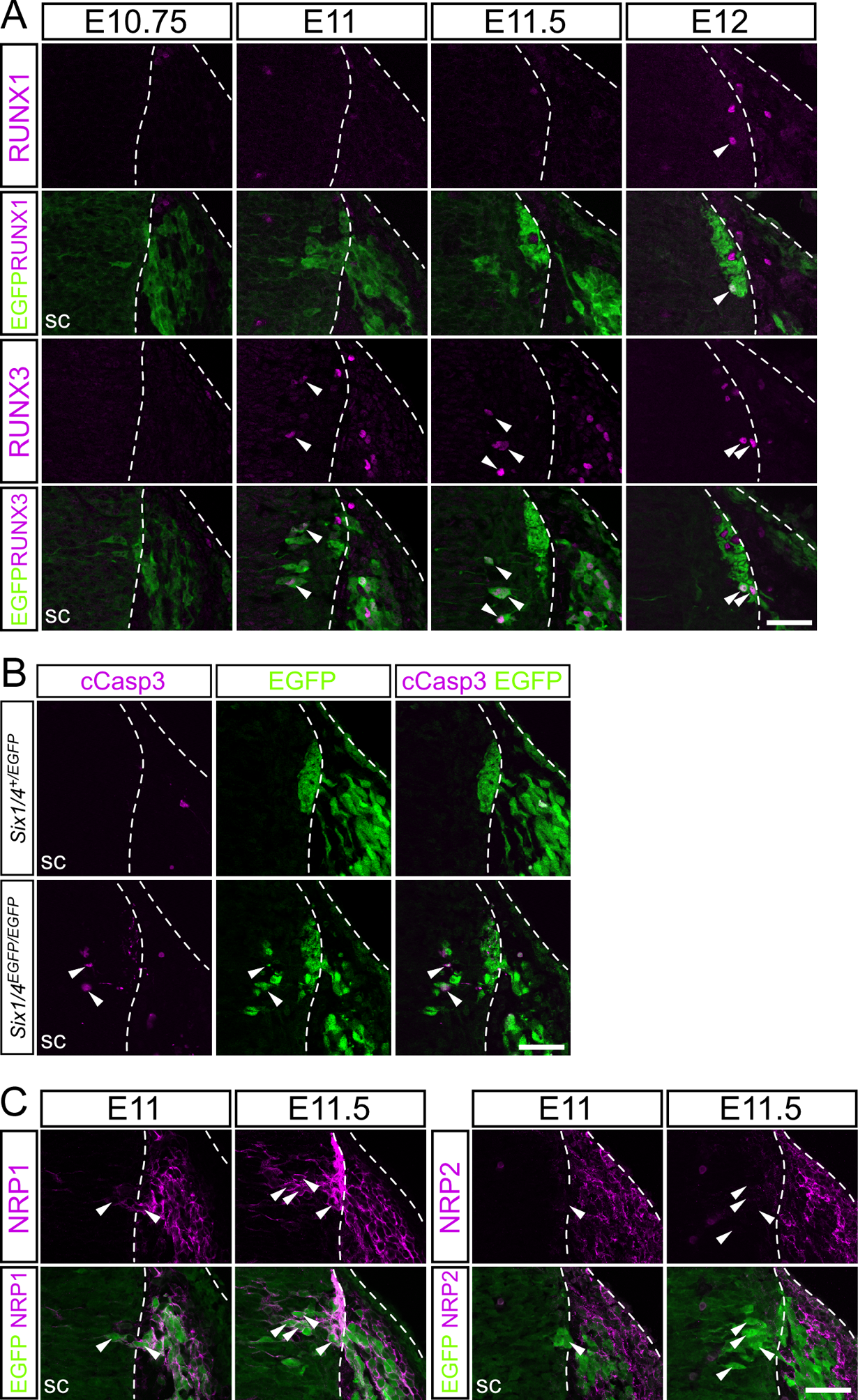

Supplement: Additional file 4 — Intramedullary EGFP-positive cells in Six1/4 EGFP/EGFP mice are positive for RUNX1, RUNX3 cleaved CASP3 and NRP1. (A) The majority of intramedullary EGFP-positive cells in Six1/4 EGFP/EGFP embryos (green in spinal cord (sc)) are positive for RUNX1 (magenta) and RUNX3 (magenta), as pointed out with arrowheads. Top line shows embryonic days of embryos. RUNX3 is detected at E11, one day earlier than that of RUNX1. (B) A substantial number of cleaved CASP3 (cCasp3)-positive cells (magenta) are observed in the spinal cords of Six1/4 EGFP/EGFP at E12 and these cells are also positive for EGFP (green, arrowheads), whereas no such cells are observed in the Six1/4 +/EGFP spinal cord. (C) The majority of intramedullary EGFP-positive cells in Six1/4 EGFP/EGFP embryos (green in sc) are positive for NRP1 (magenta), but not for NRP2 (magenta), as indicated by arrowheads. Dashed lines demarcate the position of the ectoderm and sc. Scale bars: 50 μm. [file 1741-7007-12-40-S4.tiff]

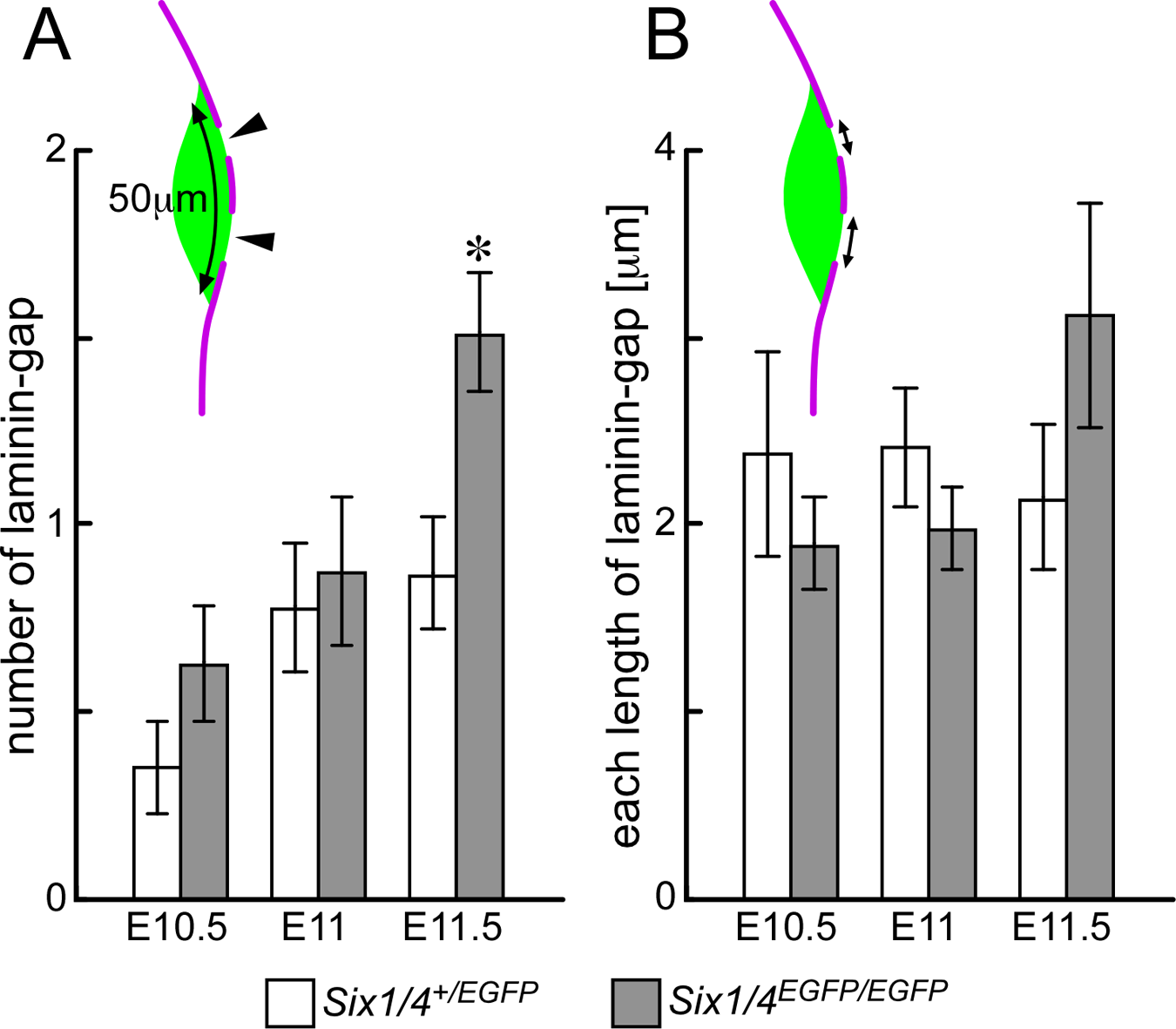

Supplement: Additional file 6 — Number but not length of individual laminin gaps is increased in Six1/4 EGFP/EGFP DREZ. (A) Number and (B) length of individual laminin gaps in 50 μm length of basal lamina covering the primordium of the dorsal funiculus. For all measurements, at least five sections from three embryos were used per genotype. Data are mean ± standard error of the mean. *p <0.005. [file 1741-7007-12-40-S6.tiff]
